# Acetylation-mediated regulation of ALV viral proteins: Implications for retroviral inhibition

**DOI:** 10.1371/journal.ppat.1014229

**Published:** 2026-05-18

**Authors:** Ning Cui, Xiaoxia Han, Qinghua Huang, Peipei Wang, Lili Wang, Chuantian Xu, Shuai Su

**Affiliations:** 1 Institute of Animal Science and Veterinary Medicine, Shandong Academy of Agricultural Sciences, Jinan, China; 2 Key Laboratory of Livestock and Poultry Multi-omics of MARA, Jinan, China; 3 College of Life Sciences, Shandong Normal University, Jinan, China; 4 Tiankang Bio-pharmaceutical Co., LTD, Wulumuqi, China; 5 Shandong Provincial Key Laboratory of Animal Biotechnology and Disease Control and Prevention; College of Veterinary Medicine, Shandong Agricultural University, Tai’an, China; University of Illinois at Chicago College of Medicine, UNITED STATES OF AMERICA

## Abstract

Avian leukosis virus (ALV), a prototypic alpharetrovirus, serves as a pivotal model for studying retroviral biology due to its well-characterized replication cycle, genetic tractability, and evolutionary conservation with clinically relevant retroviruses like HIV. This study identified 12 novel lysine acetylation (KAc) sites in ALV proteins through mass spectrometry, revealing striking conservation across multiple ALV subgroups and distributed as follows: 3 in the matrix (MA) protein, 1 in the capsid (CA) protein, 1 in the reverse transcriptase (RT) protein, and 7 clustered in the integrase (IN) protein. Functional characterization via site-directed mutagenesis demonstrated that the majority of these sites serve as critical regulatory switches during viral replication, with individual KAc modifications at distinct sites differentially regulating ALV replication. Mechanistically, we discovered that the host acetyltransferase HAT1 physically interacts with ALV RT protein to mediate its acetylation at the evolutionarily conserved RT13K residue. This modification, which is conserved across multiple ALV subgroups and other retroviral species, significantly enhances both RT enzymatic activity and viral replication efficiency. Our findings establish KAc as a critical post-translational regulator of alpharetrovirus replication and demonstrate that RT13K acetylation is a key modulator of ALV RT function. The conservation of this site across retrovirals suggests its functional importance and warrants further investigation to explore its potential as a broad-spectrum antiviral target. These results not only deepen understanding of epigenetic regulation in viral replication but also provide a framework for developing acetylation-targeted antiretroviral strategies.

## Author summary

Avian leukosis virus (ALV) serves as a valuable model for studying retroviral replication. Although antiretroviral therapies exist, challenges remain in achieving complete viral control, and a better understanding of host-virus interactions is needed. In this study, we identified 12 novel KAc sites in ALV proteins that are conserved across multiple ALV subgroups. Using site-directed mutagenesis, we found that individual acetylation events differentially regulate viral replication. Mechanistically, we discovered that the host acetyltransferase HAT1 interacts with ALV RT protein and acetylates the conserved RT13K residue, which significantly enhances RT activity and viral replication. Our findings establish KAc as a critical regulator of retroviral replication and suggest that the conserved RT13K site may represent a potential broad-spectrum antiviral target. These insights provide a framework for developing acetylation-targeted antiretroviral strategies.

## Introduction

Avian leukosis virus (ALV), an oncogenic retrovirus of the *alpharetrovirus* genus, has emerged as a pivotal model for studying fundamental retroviral biology and pathogenesis. This virus primarily infects poultry, causing immunosuppression, growth retardation, and diverse neoplastic conditions [1–4]. Its broad range of envelope subgroups—specifically A, B, C, D, E, J, and K in chickens, and F, G, H, and I in pheasants, partridge, and quail—makes it an excellent system not only for investigating retroviral entry mechanisms but also for studying viral evolutionary dynamics [5]. The recently emerged ALV-K subgroup exemplifies retroviral genetic plasticity, demonstrating unique evolutionary trajectories through pol gene deletions that enhance replication efficiency [6] and inter-subgroup recombination events acquiring env segments and LTR regions [7,8]. Notably, ALV-K has become dominant in farms implementing ALV-J eradication programs [9–11], showcasing remarkable retroviral adaptation under selective pressure. Its characteristic subclinical infections with growth impairment and immune atrophy [12,13], along with frequent asymptomatic carriage [9–11], provide valuable insights into retroviral strategies for persistent infection and latency establishment. These features, combined with its distinct superinfection interference patterns [5,14] and host-specific adaptation, position ALV-K as an indispensable model for understanding broader retroviral phenomena, from HIV persistence to HTLV-mediated oncogenesis, while also representing a significant economic burden on global poultry production [15–20].

During viral infection, both host and viral proteins undergo extensive post-translational modifications (PTMs), including phosphorylation, ubiquitination, methylation, and acetylation, which critically regulate viral replication, pathogenesis, and host antiviral responses [21–26]. Among these, protein lysine acetylation (KAc) stands out as one of the earliest discovered and most functionally significant PTMs. Although KAc was first identified over six decades ago, its functional characterization remained limited until recent years, when advances in proteomics and molecular biology spurred a surge in research [27]. KAc is now recognized as a key regulator of diverse biological processes, including transcriptional regulation, metabolic reprogramming, and protein stability. This reversible and dynamic modification is catalyzed by lysine acetyltransferases (KATs), which transfer acetyl groups from acetyl-CoA to substrate proteins, while lysine deacetylases (KDACs) mediate its removal. The human genome encodes 17–22 classical KATs and 18 KDACs, which orchestrate the dynamic and finely tuned regulation of protein acetylation [28]. Despite extensive studies on host protein acetylation, only a limited number of viral proteins have been confirmed to undergo KAc modification. To date, KAc has been reported in proteins from Bombyx mori Nucleopolyhedrovirus (BmNPV), human *Papillomavirus* (HPV), influenza A virus (IAV), human immunodeficiency virus type 1 (HIV-1), severe acute respiratory syndrome coronavirus (SARS-CoV), hepatitis delta virus (HDV), and Ebola virus (EBOV) [29–38]. Among these, IAV exhibits the highest number of experimentally validated KAc sites, with modifications identified in nucleoprotein (NP), non-structural protein 1 (NS1), polymerase acidic protein (PA), and Matrix protein 2 (M2) proteins. Notably, mutagenesis studies have demonstrated that KAc sites in NP, NS1, and PA significantly influence IAV replication efficiency [39–42]. HIV-1, another retrovirus like ALV, also undergoes KAc modifications—particularly in the trans-activator of transcription (Tat) and integrase (IN) proteins—during its transcriptional and replicative cycles [32,33,43,44]. Nevertheless, the biological consequences of HIV-1 protein acetylation continue to be debated, particularly regarding the impact of KAc in the IN C-terminal domain (CTD) on viral replication. Currently, there are no documented investigations into the functional roles of protein acetylation in other retroviral systems, highlighting a critical gap in our understanding of how acetylation regulates retrovirus biology.

Recent years have witnessed remarkable progress in protein acetylation research, largely propelled by technological innovations in mass spectrometry and antibody-based enrichment methodologies. The advent of high-throughput acetylomics has revolutionized the field, enabling comprehensive mapping of acetylation sites across both viral and host proteomes. This breakthrough provides unprecedented opportunities for systematically elucidating molecular mechanisms underlying viral pathogenesis. Notable examples include the acetylomic profiling of cells infected with BmNPV and IAV, which identified critical acetylation modifications on the viral proteins [30,37]. Building upon these technological advancements, our research team has made significant contributions to the field by conducting the first comprehensive acetylproteomic analysis of *alpharetrovirus* ALV-K strain JS11C1. Through this pioneering work, we have successfully identified novel KAc sites in retoviral proteins, followed by systematic functional characterization of these modification sites. These findings substantially expand our understanding of post-translational regulation in retroviruses.

## Results

### Unraveling the acetylation landscape of the *alpharetrovirus* ALV proteins during infection

**Fig 1:**
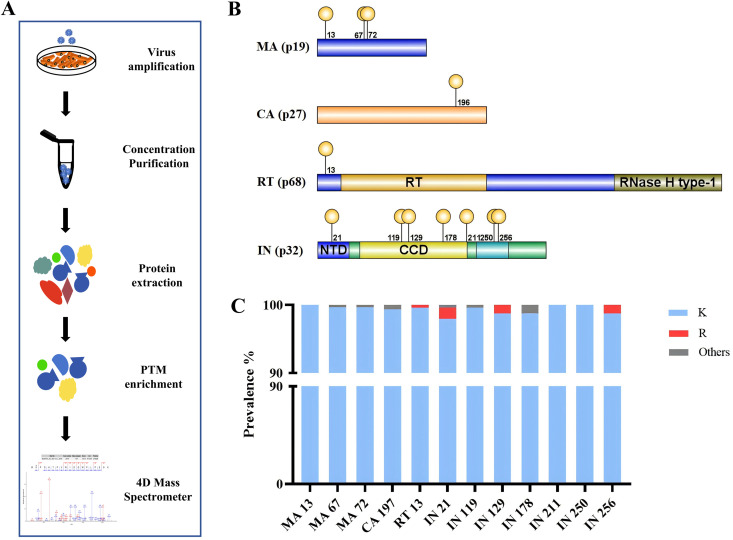
Identification and characterization of KAc sites in ALV proteins. **(A)** Experimental workflow for the identification of KAc sites in ALV viral proteins. **(B)** Distribution of identified KAc sites across the domains of each viral protein. **(C)** Evolutionary conservation analysis of the identified KAc sites among *alpharetrovirus* ALV proteins.

To precisely map KAc sites in ALV proteins during avian cell infection, we purified virions from the culture supernatant of infected DF1 cells and performed comprehensive Liquid chromatography-tandem mass spectrometry (LC-MS/MS) analysis (Fig 1A). Our proteomic investigation revealed 12 novel KAc sites distributed across multiple viral proteins (Fig 1B), with the following distribution: 3 acetylation sites in the matrix (MA) protein, 1 site each in the capsid (CA) and reverse transcriptase (RT) proteins, 7 sites clustered in the IN protein. The complete protein sequence coverage data for all virus-host combinations are provided in S1 Table, while representative fragment spectra for each identified acetylation site are presented in S1-S12 Figs. Subsequent bioinformatic analysis of ALV sequences from the National Center for Biotechnology Information (NCBI) Genbank database demonstrated that these modified lysine (K) residues exhibit remarkable evolutionary conservation across diverse avian ALV strains (Fig 1C).

To assess the spatial distribution of the identified acetylation sites, we generated three-dimensional structure models of ALV proteins using homology modeling (SWISS-MODEL) and AlphaFold 3. For MA, homology modeling using PDB 5KZ9.1 (98.06% sequence identity) revealed that K13 and K72 are located within α-helices, whereas K67 is in a coil region. AlphaFold 3 predicted that acetylation at K72 introduces a new hydrogen bond with Ser (S) 68 (S13 Fig). For CA, homology modeling using PDB 5A9E.1 (96.65% sequence identity) indicated that K196 aligns with K353 in the reference structure and may stabilize a loop region. AlphaFold 3 predicted that acetylation at this site results in loss of a hydrogen bond with Ala (A) 154 (S14 Fig). For RT, homology modeling using PDB 7SR6.1 (38.52% sequence identity) showed that K13 aligns with K50 in the HERV-K RT ternary complex. AlphaFold 3 generated a reliable model (pIDDT > 90 for most regions). Acetylation at K13 was predicted to disrupt the α-helical structure, narrowing the nucleic acid-binding groove (S15 Fig). For IN, homology modeling based on the RSV transfer complex cryo-EM structure (98.92% sequence identity) revealed that the acetylation sites are distributed across multiple IN-derived polypeptides (S16 Fig). All sites form varying numbers of hydrogen bonds with neighboring residues. Notably, K119 is predicted to form a hydrogen bond with the viral nucleic acid chain (6.61 Å), and K129 forms another with I/DG`31 (5.61 Å), suggesting a role in stabilizing the DNA-IN interaction.

### The impact of acetylation sites on ALV replication

**Fig 2:**
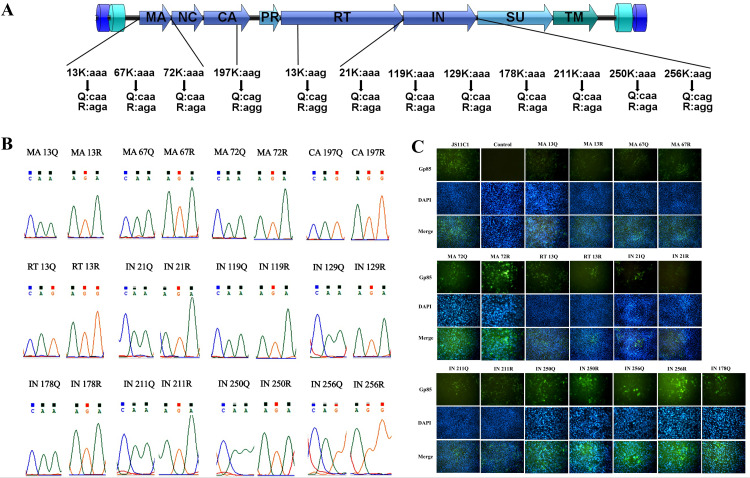
Construction and verification of point-mutated viruses. **(A)** PCR-based site-directed mutagenesis schematic. Wild-type and mutant residues are shown under the ALV genomic structure. **(B)** Sequencing chromatograms confirming the successful introduction of the point mutation. **(C)** IFA verification for the rescued parental virus, mutant viruses and mock-infected control cells. DF-1 cells were infected with the indicated viruses at an MOI of 0.01. At 48 hpi, cells were stained with mouse polyclonal anti-gp85 antibody (1:100 dilution, green) and counterstained with DAPI (blue) for nuclear visualization.

To assess the role of each acetylation site identified in the viral proteins of ALV virions, we generated mutant viruses based on the infectious clone of JS11C1 from our previous study. At each site, we introduced arginine (R) substitution to mimic the non-acetylated state and glutamine (Q) substitution as a complementary probe for acetylation mimicry. Because Q substitution has inherent limitations as a surrogate for acetyl-lysine, our functional interpretation focused primarily on the differential effects between the R and Q mutants (Fig 2A). Site mutant infectious clones were confirmed by sequencing the viral genes (Fig 2B) and rescued wild type (WT) mutant viruses were verified by immunofluorescence assay (IFA) using mouse polyclonal anti-gp85 (Fig 2C), respectively. Finally, we could generate 8 pairs of mutant viruses including MA-K13Q, MA-K13R, MA-K67Q, MA-K67R, MA-K72Q, MA-K72R, RT-K13Q, RT-K13R, IN-K21Q, IN-K21R, IN-K211Q, IN-K211R, IN-K250Q, IN-K250R, IN-K256Q, and IN-K256R (Fig 2C), respectively. However, we failed to generate mutant viruses for acetylation sites at CA196, IN119, IN129, or IN178 after at least three rounds of transfection, among them, only IN-K178Q could be rescued with relatively low viral titer. Therefore, to study the effects of viral protein acetylation on ALV infections, we narrowed our focus to the 8 pairs of mutant viruses for further analysis.

**Fig 3:**
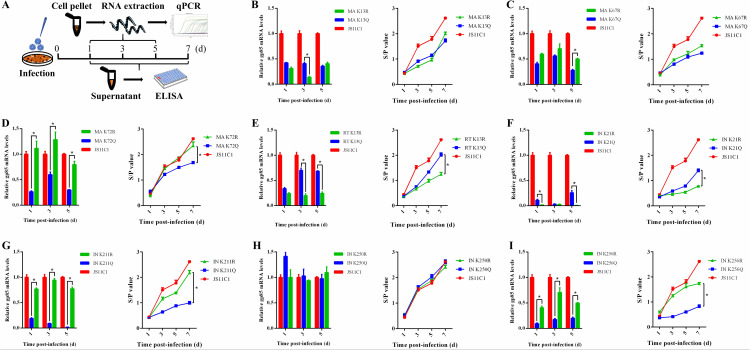
KAc site mutation effects on ALV transcription and production. **(A)** Schematic diagram of sample collection and processing. The viruses were inoculated into DF-1 cells at an MOI of 0.01, and the virus-containing supernatants were collected at the indicated time points for qRT-PCR and ELISA. (B-I) gp85 transcription and p27 expression of ALV mutant viruses compared to parental virus detected by RT-qPCR and ELISA. Data presented as mean ± SEM are from three independent experimental replicates.

In order to characterize the growth properties of the 8 pairs of mutant viruses, DF1 cells were infected with WT JS11C1 and these mutant viruses at an MOI of 0.01, respectively. Cell lysate were detected for ALV gp85 transcription at 1, 3, and 5 days post-infection (dpi), and culture supernatant were detected for ALV p27 expression at 1, 3, 5, and 7 dpi (Fig 3A). For MA protein, both MA13 (Fig 3B) and MA67 (Fig 3C) mutant viruses, displayed comparative decreased growth capacity compared to WT JS11C1, while there was no significant differences between the mimic acetylation types (K → Q or K → R), suggesting that the acetylation modification at these sites have little or no effect on viral replication and the impaired viral replication may be caused by conformational changes or other structural modifications of MA protein. MAK72R mutant virus displayed similar replication capacity compared with the WT JS11C1, while viral growth of MAK72Q mutant virus was significantly impaired (Fig 3D), suggesting that the impaired replication of the MAK72Q mutant may be attributable to non-specific effects of the Q substitution rather than directly mimicking acetylation at this residue. On the contrary, compared to WT JS11C1 and RTK13Q, viral growth of RTK13R mimic non-acetylation was significantly impaired, suggesting a positive role for the acetylation at RT13 (Fig 3E). For IN protein, the acetylation modification at IN21 (Fig 3F) showed similar effect as RT13, while the acetylation modification at IN211 (Fig 3G) and IN256 (Fig 3I) showed similar effect as MA72. Viral replication was unaffected, regardless of whether at the transcriptional or translational level, by either Q substitution or R substitution at IN250 (Fig 3H), indicating that acetylation at IN250 is likely non-functional for viral replication. Taken together, the RT K13R mutant exhibited impaired replication compared to RT K13Q, and a similar phenotype was observed for the IN K21R mutant, suggesting a positive regulatory role for the dynamic acetylation/deacetylation cycle at these sites. In contrast, mutations at MA72, IN211, and IN256 displayed distinct replication phenotypes, with the Q mutants showing more pronounced defects than the corresponding R mutants.

### Role of host acetyltransferases in ALV replication

**Fig 4:**
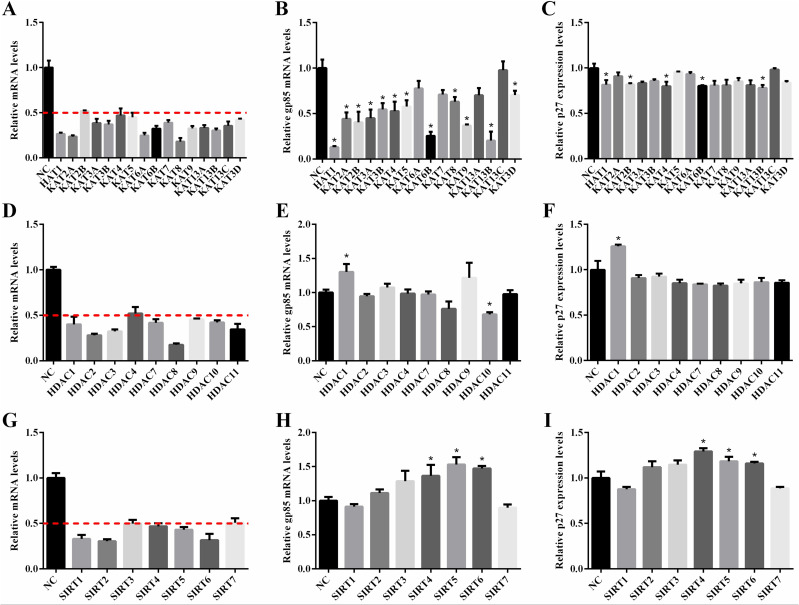
Candidate protein knockdown effects on ALV replication. (A, D, **G)** The silencing efficiency of the siRNAs target to the candidate protein. The DF1 cells were transfected with siRNAs with a final siRNA concentration of 50 nM for 24h, and the mRNA level of candidate protein was detected by RT-qPCR. (B, C, E, F, H, **I)** Silencing candidate protein effects on virus replication. DF1 cells were transfected with the individual siRNA targeted to a protein for 24 h then infected with ALV. After 24 h of infection, the cells were collected to determine the gp85 transcription by RT-qPCR (B, E, H) and the virus-containing supernatants were collected for ELISA detection (C, F, **I)**. Data presented as mean ± SEM are from three independent experimental replicates.

To test the role of host acetyltransferases in *alpharetrovirus* ALV replication, small interfering RNAs (siRNAs) were synthesized to knock down the expression of acetylases (HAT family) and deacetylases (HDAC and SIRT families) in DF1 cells. RNA interference (RNAi) results showed that nearly all HATs, HDACs, and SIRTs family proteins was 50% lower than the control group at 24h after transfecting siRNAs (Fig 4A, 4D, and 4G). Then, we tested the role of HATs, HDACs, and SIRTs family proteins on the gp85 transcription and p27 expression at 48 hours post-infection (hpi). Knockdown of HAT1, KAT2B, KAT4, KAT6B, or KAT13B significantly reduced both of the transcription of gp85 and expression of p27 (Fig 4B and 4C), whereas knockdown of HDAC1 (Fig 4E and 4F), SIRT4, SIRT5, or SIRT6 (Fig 4H and 4I) markedly enhanced both processes. Hence, we concluded that acetylation modification might play a positive regulatory role in ALV replication, in that HAT-mediated acetylation and HDAC1/SIRT4/5/6-dependent deacetylation might coordinately regulate protein acetylation to affect viral replication efficiency.

Among the examined host acetyltransferases, HAT1 knockdown exerted the most pronounced effect on gp85 transcript levels. To evaluate whether HAT1 knockdown exerts nonspecific effects on cellular homeostasis, cell viability and apoptosis were assessed in parallel (S17 Fig). CCK-8 assay revealed that at the time point corresponding to viral replication analysis (24 hpi, equivalent to 48 h post-transfection), the relative cell viability in the HAT1 knockdown group was 96.45% of that in the control group, with no statistically significant difference detected (P > 0.05). Annexin V/PI flow cytometry analysis demonstrated a marginal increase in apoptotic cell populations following HAT1 knockdown: early apoptotic cells increased from 2.25% in the NC siRNA control group to 4.14%, and late apoptotic cells increased from 3.47% to 6.64%. However, these differences did not reach statistical significance (P > 0.05 for both comparisons). Collectively, these data indicate that HAT1 knockdown does not significantly compromise cell viability or induce substantial apoptosis under the experimental conditions examined.

### Acetylation at RT13K promotes viral replication by enhancing the formation of infectious virus particle and RT activity

**Fig 5:**
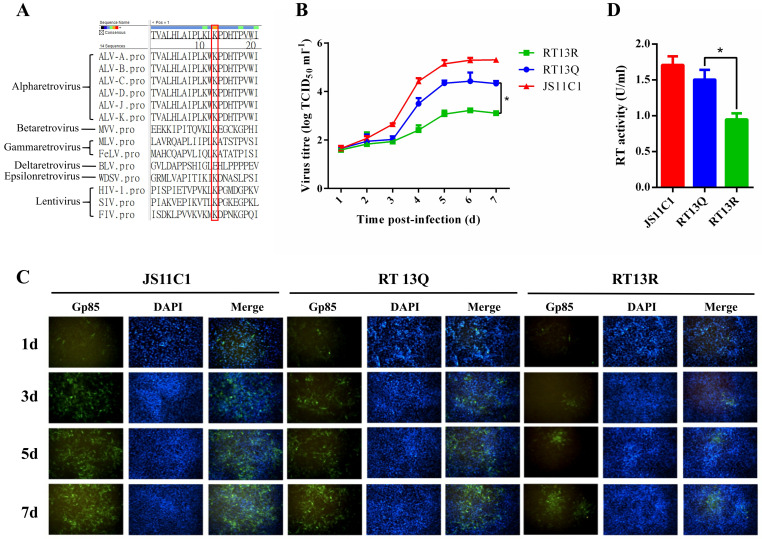
Conservation and functional analysis of the RT K13 residue. **(A)** Conservation of RT K13 across retrovirus species. **(B)** Growth curve of WT JS11C1 virus versus mutant viruses. The viruses were inoculated into DF-1 cells at an MOI of 0.01, and the virus-containing supernatants were collected at the indicated time points for virus titration. Data presented as mean ± SEM are from three independent experimental replicates. **(C)** RT activity quantification of wild-type versus mutant viruses. Data presented as mean ± SEM are from three independent experimental replicates. **(D)** Time-course IFA analysis of WT JS11C1 virus and mutant viruses infection. Representative IFA images of DF1 cells infected with WT JS11C1 virus or mutant viruses at 1d, 3d, 5d, and 7 dpi. Viral antigen (green, anti-gp85 mAb at 1:100 dilution), nuclei (blue, DAPI).

Analysis of viral replication through knock down of host acetyltransferase revealed that acetylation likely plays a positive regulatory role in ALV replication. This finding is phenotypically consistent with the aforementioned results that RT13 and IN21 exerted positive effects on viral replication. Specially, RT13K was the only acetylated site in RT protein, and the sequence alignment demonstrates strong evolutionary conservation of K13 in RT protein across multiple ALV subgroups (Fig 1C) and other retrovirus species, suggesting essential functional roles in the retroviral life cycle (Fig 5A). Therefore, we then systematically investigated the effect of RT13K acetylation on viral replication dynamics. The multi-step growth results demonstrated that the viral titer of mutant RTK13Q virus was significantly higher than RTK13R starting from 3 dpi (Fig 5B). Intracellular tracing of viral gp85 protein expression revealed that the number of infected cells were consistent with the multi-step growth results (Fig 5C). It is interesting to find that mutant RTK13Q virus maintained a replication pattern similar to WT JS11C1 virus, with diffuse distribution throughout the culture system, while RTK13R virus-infected cells showed obvious aggregation, demonstrating alterations in viral transmission patterns. Furthermore, RT activity assays indicated significantly reduced enzymatic activity in RTK13R compared to WT JS11C1 virus or RTK13Q (Fig 5D), which represents another crucial factor contributing to its attenuated replication. Collectively, the results demonstrated that RT13K acetylation may affect viral replication by altering the virus’s cell-to-cell transmission and RT activity.

### HAT1 interacts with RT and catalyzes its acetylation

**Fig 6:**
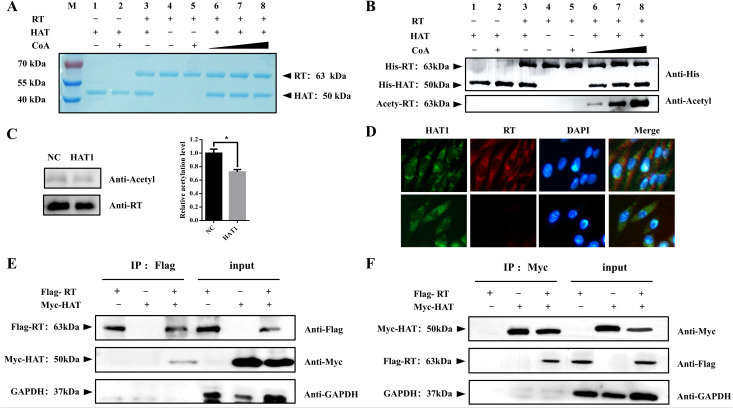
HAT1 interacts with and acetylates the RT protein. **(A, B)** *In vitro* acetylation assay. Purified RT protein was incubated with varying concentrations of acetyl-CoA (0.1, 0.5, and 1 μM) in the presence of purified HAT1 or control buffer at 37°C for 10 min. Acetylated K residues were detected by Western blotting with an anti-acetyl-lysine antibody **(B)**. Coomassie Blue staining shows input protein levels **(A)**. **(C)** Acetylation of RT during viral infection. DF1 cells were infected with the JS11C1 virus, with or without HAT1 knockdown. Protein lysates were collected at 3 dpi, and IP was performed using an anti-RT antibody, followed by immunoblotting with an anti-acetyl-lysine antibody. **(D)** Co-localization of HAT1 and RT during infection. DF1 cells infected with JS11C1 virus were subjected to IFA at 48 hpi using anti-HAT1 (1:100 dilution) and anti-RT (1:250 dilution) antibodies. **(E)** Forward co-IP. Lysates from HEK-293T cells co-expressing Flag-RT and Myc-HAT1 were immunoprecipitated with anti-Flag beads, followed by immunoblotting with anti-Flag (top) or anti-Myc (bottom). **(F)** Reverse co-IP. Reciprocal immunoprecipitation was performed using anti-Myc beads, followed by immunoblotting with anti-Myc (top) or anti-Flag (bottom).

To investigate the acetylation catalytic effect of HAT1 on the RT protein, pET30a-His-HAT1 and pET30a-His-RT were transformed into *E. coli* BL21(DE3), followed by protein expression induction and purification. Highly purified HAT1 and RT proteins were obtained, with purity exceeding 90% (Fig 6A). *In vitro* HAT acetylation assays revealed that the acetylation level of the RT protein increased in a concentration-dependent manner with increasing acetyl-CoA, confirming that the purified HAT1 possesses acetylation catalytic activity and can directly acetylate the RT protein (Fig 6B).

To further validate whether RT undergoes acetylation under physiological conditions, co-immunoprecipitation (co-IP) assays were performed using protein lysates collected at 3 dpi. Immunoprecipitation was conducted with an anti-RT antibody, followed by immunoblotting with an anti-acetyl-lysine antibody. The results demonstrated that RT is indeed acetylated during viral infection *in vivo*. Notably, upon knockdown of HAT1, the acetylation level of RT was significantly reduced (P < 0.05) (Fig 6C).

To elucidate the mechanism by which HAT1 catalyzes RT acetylation, IFA and co-IP assays were conducted. IFA performed at 48 hpi revealed that HAT1 and RT co-localize in both the cytoplasm and the nucleus (Fig 6D). For co-IP analysis, pcAGGS-HAT1-Myc and pCAGGS-Flag-RT plasmids were transfected either individually or in combination into HEK-293T cells. Protein lysates were harvested at 24 h post-transfection, and reciprocal co-IP assays were performed using anti-Flag and anti-Myc antibodies. The results confirmed an interaction between HAT1 and the RT protein (Fig 6E and 6F). Collectively, these findings suggest that HAT1 directly interacts with RT and catalyzes its acetylation.

## Discussion

Currently, KAc modifications have been specifically documented only for the Tat and IN proteins within the HIV-1 among the retroviral family [32,33,43]. For other retroviruses, comprehensive investigations of analogous PTMs remain lacking. In this study, we focused on the functional significance of KAc in *alpharetrovirus* ALV-K by systematically mapping KAc sites across the viral proteome using LC-MS/MS. Consistent with observations in HIV-1 IN, the integrase protein of ALV is also subject to acetylation [32,33]. Strikingly, *alpharetrovirus* ALV IN exhibits the highest number of acetylation sites among viral proteins, implying a potential key regulatory role of this modification in retroviral integrase activity, including DNA binding, endonucleolytic cleavage, strand transfer and even oligomerization. Unlike HIV-1, which encodes the Tat protein as a critical transcriptional transactivator, ALV transcription depends predominantly on host cellular machinery, including RNA polymerase II and endogenous promoter/enhancer elements, without the need for a Tat-like viral regulator [45,46]. Notably, our study also revealed acetylation modifications on other ALV proteins including MA, CA, and RT, validating the sensitivity and reliability of our KAc site identification platform for virus proteins. Sequence conservation analysis reveals that these modified K residues are highly conserved across diverse avian ALV strains, further validating the use of ALV-K as a representative research model. Nevertheless, extrapolating these findings to other viral subgroups or species will require direct experimental validation. Overall, this discovery not only demonstrates the robustness of our methodology in identifying previously unrecognized modification sites but also significantly broadens the landscape of acetylation research in retroviral proteins.

Through reverse genetics and site-directed mutagenesis, we successfully rescued mutant viruses carrying Q substitutions as complementary probes for acetylation mimicry, or R substitutions to mimic the non-acetylated state at the identified lysine residues. Most of these mutants were viable, suggesting that KAc at these positions is not essential for basic viral replication but may fine-tune specific stages of the ALV-K life cycle. However, we failed to rescue mutant viruses for KAc sites at CA196, IN119, IN129, or IN178 after at least three rounds of transfection, suggesting that they might be crucial for virus fitness. For instance, The conservation of several acetylated K sites across retroviral IN and their spatial positioning near functional domains (e.g., nucleic acid-binding interfaces) hints at a regulatory role in processes such as strand transfer complex formation [47]. the acetylation-mimetic mutation at IN119 and IN129—proximal to viral DNA in the stable transfer complex—may perturb DNA-binding affinity, akin to observations in HIV-1 IN [33,48]. Our reverse genetics analysis revealed that all identified KAc sites, except IN K250, impacted viral replication to varying extents when mutated to either Q or R substitutions. This striking phenotypic consistency across multiple sites strongly supports the broad functional importance of KAc in regulating retrovirus replication [32,43,44,49]. The singular exception at IN K250 suggests this residue may occupy a structurally permissive position where charge modulation is tolerated. Acetylation at distinct sites of the same protein can produce entirely different functional outcomes [33,39]. In IN protein, acetylation at IN21, IN211, and IN256 directly affected viral replication, while acetylation at IN250 had only a weak effect. Thus, acetylation at different sites exerts distinct impacts on ALV replication, ranging from strong to minimal. This “acetylation code” may fine-tune viral gene expression by integrating multiple cellular signals. Further studies are needed to elucidate the underlying mechanisms, but these findings establish ALV as a valuable model for studying how PTMs regulate the retroviral life cycle.

ALV, a retrovirus that has plagued the poultry industry for over a century [1,2], continues to cause substantial economic losses [2,3]. Conventional control measures face significant challenges, necessitating novel antiviral strategies [2]. Recent advances highlight the pivotal role of epigenetic regulation in virus-host interactions [26,28,29,50]. Notably, HDAC inhibitors have emerged as broad-spectrum antivirals [51,52], yet the specific contributions of acetylation-modifying enzymes to ALV replication remain poorly understood. In this study, we systematically investigated the impact of host acetylation machinery on ALV-K replication. RNAi experiments revealed that acetyltransferases HAT1, KAT2B, KAT4, KAT6B, and KAT13B promoted viral replication, whereas deacetylases HDAC1, SIRT4, SIRT5, and SIRT6 exerted antiviral effects. Although HAT1 knockdown caused mild reductions in cell viability and a slight increase in apoptosis, the effects on viral replication were disproportionately larger: gp85 mRNA decreased by over 50% and p27 protein by approximately 20%, compared to only a 3.55% reduction in cell viability. This disparity suggests that the antiviral effect of HAT1 knockdown involves specific interference with viral life cycle processes rather than nonspecific cytopathic changes [53–55]. Notably, we demonstrate that HAT1 physically interacts with and acetylates ALV RT at the evolutionarily conserved K13 residue, which significantly enhances RT activity and viral replication. As shown in Figs 1C and 5A, K13 is highly conserved across all major ALV subgroups and other retroviral genera, suggesting that this acetylation site may serve as a potential broad-spectrum anti-retroviral target [53,56,57]. These results establish host acetylation enzymes as key regulators of ALV replication and identify the host acetylome as a druggable target for antiviral intervention.

RT protein is a characteristic enzyme in all retroviruses, with polymerase and RNase H activities, and is essential for retrovirus replication [58]. Upon retrovirus infection, the single-stranded RNA genome is converted to double-stranded DNA by the RT. After reverse transcription is completed, RT forms a complex with viral DNA, which is subsequently integrated into the host cell genome by the IN [59]. The RT activity assay results demonstrated that wild-type RT13K exhibited the highest activity, while the RTK13Q mutant virus showed significantly higher RT activity compared to the RTK13R mutant virus. This suggests that the dynamic acetylation/deacetylation cycle at K13, rather than the static acetylated or non-acetylated state alone, may enhance RT activity and promote ALV-K replication by altering protein structure and function. Structural prediction indicates that acetylation at this site disrupts the α-helical structure, leading to narrowing of the nucleic acid-binding groove near the catalytic core. Our results are consistent with Weiss et al. (2000) that local conformational changes in the RT active site determine its catalytic function. Mutations in the β8-αE loop altered HIV-1 RT processivity. Although those mutations mostly reduced processivity, our K13Q mutant enhanced RT activity, suggesting distinct modifications regulate enzyme function via divergent mechanisms. Thus, our data support the principle that conformation determines function and reveal that acetylation enhances reverse transcription by reshaping the substrate-binding channel [60]. Concurrently, this structural remodeling also reprograms the viral transmission mode, possibly by altering RT’s interaction with host factors involved in cytoskeleton-dependent trafficking [61]. Notably, in the virus rescue experiments, cells infected with the RT K13R mutant virus exhibited a distinct clustered distribution, whereas the WT JS11C virus and RT K13Q mutant virus displayed a more dispersed infection pattern (Fig 5C). This observation suggests that the KAc of K13 in RT may alter the cell-to-cell transmission mode of ALV-K, favoring direct cell-to-cell spread (e.g., via membrane fusion, tunneling nanotubes, or virological synapses) over the conventional extracellular release pathway. Since direct cell-to-cell transmission is generally less efficient than extracellular diffusion, this could explain the lower replication efficiency observed in the RTK13R mutant virus. Indeed, certain mutations in HIV-1 RT or Env have been shown to enhance virological synapse-dependent transmission while reducing free virus release [61–63]. Similarly, actin inhibitors can block cell-to-cell spread of MLV without affecting free virus infection [64]. These findings raise the question: Does the deacetylation of 13K in RT impair viral interactions with the cytoskeleton (e.g., actin or microtubules)? Further investigation is required to validate this hypothesis. A deeper understanding of how this residue regulates viral transmission mechanisms will not only shed light on novel pathogenic mechanisms of ALV but also provide a theoretical foundation for developing antiviral therapies targeting cell-to-cell viral spread.

## Conclusions

**Fig 7:**
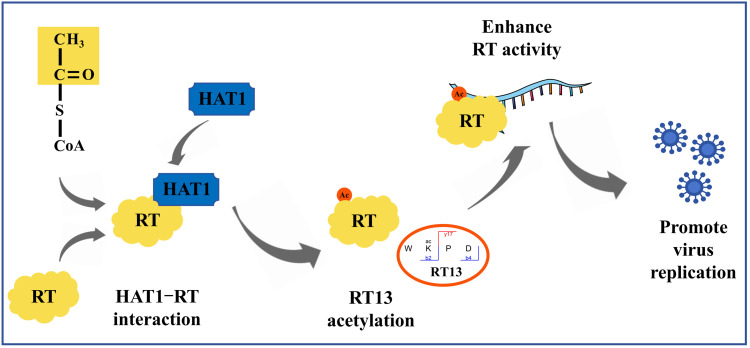
Schematic summary of HAT1-mediated acetylation of RT protein enhancing RT activity to promote ALV replication.

This study provides the first comprehensive identification of KAc modification sites in multiple *alpharetrovirus* ALV viral proteins. Through systematic construction of mutants designed to disrupt or mimic reversible acetylation at specific K residues, we functionally validated that the majority of these sites serve as critical regulatory switches during viral replication. Further investigation revealed that HAT1 physically interacts with RT and mediates its acetylation. Specifically, acetylation at the highly conserved RT13K residue augmented its RT enzymatic activity, which consequently enhances viral replication efficiency (Fig 7). Collectively, our work uncovers the functional significance of viral protein acetylation in retroviral replication, provides tools for epigenetic virology research, and opens new avenues for antiretroviral therapy development.

## Materials and methods

### Cells and viruses

The HEK293T and DF-1 cell lines were purchased from Procell Life Science & Technology Co., Ltd., China. Both cell lines were cultured in Dulbecco’s Modified Eagle’s Medium (DMEM; Gibco) supplemented with 10% fetal bovine serum (FBS; Gibco), and maintained at 37°C in a humidified 5% CO₂ atmosphere. The ALV-K strain JS11C1 (GenBank accession no. KF746200) was originally isolated in 2012 by College of Veterinary Medicine, Shandong Agricultural University, from a liver tumor of a Chinese Luhua chicken, a native breed [14].

### Plasmid construction

The ALV-K infectious clone was constructed by inserting the proviral DNA of ALV-K isolate JS11C1 (GenBank accession no. KF746200) into the pBluescript II KS(+) backbone, with site-directed mutagenesis performed to generate mutant clones using primers listed in S2 Table. The gallus HAT1 CDS was cloned into pCAGGS with an N-terminal Myc tag (pCAGGS-HAT1-Myc), while the ALV RT gene was similarly cloned into pCAGGS with an N-terminal Flag tag (pCAGGS-Flag-RT). Additionally, both HAT1 and RT were subcloned into pET30a with N-terminal His tags (pET30a-His-HAT1 and pET30a-His-RT, respectively) for recombinant protein expression and purification.

### Virus purification

Viruses grown in cell culture were harvested from the growth media of four T175 flasks of infected DF1 cells (120 ml; 10^5^ TCID_50_/ml of virus for JS11C1) at 5 dpi. The medium was clarified by low-speed centrifugation (4000 g/10 min at 4℃), then add to an Amicon Ultra-15 ultrafiltration tube and centrifuge at 4000 g/10 min at 4℃ to concentrate the virus. The concentrated virus was diafiltrated 10 fold to buffer (20 mM Tris, 150 mM sodium chloride, pH 7.5) and subjected to Capto Core 700 affinity chromatography (Cytiva) for purification to remove contaminating high abundance host proteins.

### LC-MS/MS

Briefly, samples of purified virus were reduced with 5 mM dithiothreitol for 30 min at 56 ℃ and alkylated with 11 mM iodoacetamide for 15 min at room temperature in darkness. Ttrypsin was added at 1:50 trypsin-to-protein mass ratio for the digestion overnight and the peptides were desalted by C18 SPE column. To enrich acetylation peptides, tryptic peptides dissolved in NETN buffer (100 mM NaCl, 1 mM EDTA, 50 mM Tris-HCl, 0.5% NP-40, pH 8.0) were incubated with pre-washed antibody beads (PTM101, PTM Bio) at 4 ℃ overnight with gentle shaking. Then the beads were washed for four times with NETN buffer and twice with H_2_O. The bound peptides were eluted from the beads with 0.1% trifluoroacetic acid. Finally, the eluted fractions were combined and vacuum-dried. The resulting peptides were desalted with C18 ZipTips (Millipore) according to the manufacturer’s instructions and subjected to subsequent LC-MS/MS analysis.

The tryptic peptides were dissolved in solvent A (0.1% formic acid, 2% acetonitrile/ in water), and separated with a gradient from 6% to 24% solvent B (0.1% formic acid in acetonitrile) using a home-made reversed-phase analytical column (25-cm length, 75 μm i.d.) on an nanoElute UHPLC system (Bruker Daltonics). The separated peptides were subjected to capillary source followed by the timsTOF Pro (Bruker Daltonics) mass spectrometry. Precursors and fragments were analyzed at the TOF detector, with a MS/MS scan range from 100 to 1700 m/z. The timsTOF Pro was operated in parallel accumulation serial fragmentation (PASEF) mode. Precursors with charge states 0–5 were selected for fragmentation, and 10 PASEF-MS/MS scans were acquired per cycle. The dynamic exclusion was set to 30 s. The resulting MS/MS data were processed using MaxQuant search engine (v.1.6.15.0). Tandem mass spectra were searched against the SwissProt database (20422 entries) concatenated with reverse decoy database. Trypsin/P was specified as cleavage enzyme allowing up to 2 missing cleavages. FDR was adjusted to < 1%.

### Reverse genetics

For viral rescue, DF-1 cells were transfected with recombinant plasmids using Lipofectamine 3000 (Invitrogen) in Opti-MEM (Gibco). At 5 hpi, the transfection medium was replaced with maintenance medium (DMEM containing 2% FBS). Viral supernatants were harvested at 7 dpi and subjected to blind passage in fresh DF-1 cells for three serial passages. with successful virus rescue confirmed through IFA assay for viral protein detection, PCR amplification of specific viral sequences, and subsequent Sanger sequencing validation.

### IFA

DF-1 cells seeded in 24-well plates were infected with various rescued ALV strains at an MOI of 0.01. At 48 hpi, cells were fixed with 4% formaldehyde in PBS for 15 min at room temperature, followed by PBS washing and permeabilization with 0.1% Triton X-100 in PBS for 15 min. After blocking with 1% BSA in permeabilization buffer, cells were incubated with mouse polyclonal anti-gp85 antibody (prepared by our laboratory, 1:100 dilution), mouse polyclonal anti-RT antibody (prepared by our laboratory, 1:250 dilution), or rabbit polyclonal anti-HAT1 antibody (ABIN1859122, 1:100 dilution) at 37°C for 1 h. Following three PBS washes, cells were stained with goat anti rabbit IgG (H + L) (FITC) (ImmunoWay, 1:500), goat anti mouse IgG (H + L) (AbFluor 633) (ImmunoWay, 1:200), and DAPI (NCM) for nuclear counterstaining at 37°C for 10 min. After final washing, samples were analyzed by confocal fluorescence microscopy.

### Viral growth kinetics

DF-1 cells were seeded in six-well plates and infected with different rescued ALV strains at an MOI of 0.01. Following a 2-hour adsorption period, the inoculum was removed by PBS washing and replaced with fresh DMEM supplemented with 2% FBS. For viral growth kinetics analysis, 200 μL aliquots of culture supernatant were collected at designated time points while replenishing with equal volumes of fresh medium. Viral titers in the harvested supernatants were quantified in DF-1 cells by TCID_50_ assay using the Reed-Muench method, and the resulting growth curves were generated using GraphPad Prism 6 software.

### RNAi assay

To investigate the functional roles of host acetyltransferases and deacetylases in ALV-K replication, transient knockdown experiments were performed using chemically synthesized siRNAs targeting 16 acetyltransferases (HAT family) and 16 deacetylases (HDAC and SIRT families). A non-targeting control siRNA (NC siRNA) was included as a negative control. All siRNA sequences are listed in S3 Table. DF-1 cells were seeded in 24-well plates 24 h prior to transfection to achieve approximately 70–80% confluence at the time of transfection. siRNAs were transfected using Lipofectamine RNAiMAX (Invitrogen) according to the manufacturer’s protocol, with a final siRNA concentration of 50 nM. At 24 h post-transfection, cells were harvested for RNA extraction and subsequent reverse transcription-quantitative PCR (RT-qPCR) analysis to validate knockdown efficiency. For experiments assessing viral replication, cells were infected with ALV-K JS11C1 strain at 24 h post-transfection, and samples were collected at indicated time points for further analysis. All RNAi experiments were performed in biological triplicates with three independent replicates to ensure statistical reliability of the results.

### RT-qPCR assay

Total RNA was extracted from cells using the SPARKeasy Cell RNA Kit (Sparkjade) following the manufacturer’s instructions. RNA quality and concentration were verified by spectrophotometry before reverse transcription was performed using HiScript III RT SuperMix for qPCR (+gDNA wiper) (Vazyme) to generate cDNA templates. Quantitative PCR amplification was carried out in duplicate reactions on a LightCycler 96 system (Roche) using Taq Pro Universal SYBR qPCR Master Mix (Vazyme) with gene-specific primers (sequences provided in S4 Table). The relative mRNA expression levels were calculated using the comparative threshold cycle (2^−ΔΔCq^) method, with normalization to appropriate housekeeping genes.

### Cell viability assay

DF-1 cells were seeded in 96-well plates and transfected with HAT1 siRNA, NC siRNA, or transfection reagent alone (mock control) at a final siRNA concentration of 50 nM using RNAiMAX transfection reagent. At 24, 48, and 72 h post-transfection, cell viability was assessed using the CCK-8 assay according to the manufacturer’s instructions. Absorbance was measured at 450 nm using a microplate reader. Relative cell viability was calculated as the percentage of the control group.

### Apoptosis assay

DF-1 cells were seeded in 6-well plates and transfected as described above. At 48 h post-transfection, cells were harvested using trypsin without EDTA, washed twice with cold PBS, and resuspended in binding buffer. Apoptosis was assessed by Annexin V/PI double staining according to the manufacturer’s protocol. Stained cells were analyzed by flow cytometry. Early apoptotic (Annexin V ⁺ /PI⁻) and late apoptotic (Annexin V ⁺ /PI⁺) cell populations were quantified. All experiments were performed in triplicate.

### RT assay

The viral supernatant was sequentially clarified by centrifugation (250 × g, 10 min) to remove cell debris, followed by higher-speed centrifugation (2,000 × g, 30 min) to eliminate residual particulates. PEG solution (Mr 6,000–8,000, 30% polyethylene glycol, 1.2 M sodium chloride) was used to precipitate the virus particles from the supernatant. The obtained virus particles were measure by ELISA using the Reverse Transcriptase Assay (Roche, colorimetric) according to the manufacturer’s protocol. Each sample’s absorbance (optical density, OD) was measured with a Bio-Tek Instruments EL310 microplate autoreader at a wavelength of 405 nm. The RT activity of viral samples was calculated from ELISA OD values with reference to a concurrently established standard calibration curve.

### Prokaryotic protein expression and purification

The RT or HAT1 protein was expressed in *E. coli* BL21(DE3) competent cells transformed with 2 μL of pET30a-His-RT or pET30a-His-HAT1 plasmid and plated on LB agar containing 50 μg/mL kanamycin sulfate at 37°C overnight. A single colony was inoculated into 1 mL LB medium (with kanamycin) and grown to OD600 at 0.5 ~ 0.8, followed by scaling up to 40 mL. At OD600 of 0.8, 0.2 mM IPTG was added for 16-hour induction at 15°C. Cells were harvested by centrifugation (5,000 rpm, 2 min), and expression was verified by SDS-PAGE (100V for 10 min, then 200V until dye migration) with Coomassie staining. For purification, cell pellets were lysed by sonication in 20 mM PB (pH 7.2), 300 mM NaCl, 20 mM imidazole with 1% Triton X-100, 1 mM DTT, and 1 mM PMSF. The lysate was loaded onto Ni-IDA columns pre-equilibrated with the same buffer (without detergents), and target protein was eluted using an imidazole gradient. All fractions were analyzed by SDS-PAGE to confirm purification efficiency.

### *In vitro* acetylation assay

To detect the HAT1-catalyzed acetylation, we incubated purified RT protein with purified full-length active HAT1 in the presence of HAT buffer (50 mM Tris-HCl, pH 8.0,50 mM KCl, 5%glycerol, 0.1 mM EDTA, 1 mM dithiothreitol, 1 mM PMSF, 10 mM sodiumbutyrate), and different concentrations of acetyl-CoA (Sigma), at 37℃ for 10 min. The reaction was stopped by adding loading buffer and subjected to SDS-PAGE. Proteins were analyzed by Western blotting analysis and Coomassie blue staining.

### Co-IP assay

HEK293T Cells were plated in six-well plates or 10-cm dishes. After 24 hours, cells were transfected with the indicated plasmids using Lipofectamine 3000 (Invitrogen) and lysed with ice cold RIPA buffer. Co-IP assay was performed using anti-Flag (1:50, Cell Signaling Technology), anti-Myc antibody (1:200, Cell Signaling Technology) or mouse polyclonal anti-RT antibody (1:100, prepared by our laboratory), and the immune complexes were captured on SANTA Protein G (Santa Cruz Biotechnology). Subsequently, the beads were washed five times with ice cold lysis buffer and eluted with 2 × loading buffer. Samples were evaluated by Western blotting using the indicated antibodies.

### Western blotting

Cell pellets were lysed using RIPA Lysis Buffer (NCM) supplemented with Protease Inhibitor Cocktail (NCM). The resulting lysates were mixed with reducing SDS loading buffer, denatured at 95°C for 5 minutes, and subsequently resolved by SDS-PAGE using a protein marker (Vazyme CAT: MP102). Following electrophoresis, the separated proteins were transferred onto nitrocellulose membranes. For immunodetection, the membranes were first blocked with 5% (w/v) skim milk in Tris-buffered saline containing 0.1% Tween-20 (TBST) to prevent nonspecific binding. The blocked membranes were then incubated with appropriate primary antibodies overnight at 4°C, followed by species-matched horseradish peroxidase (HRP)-conjugated secondary antibodies. Protein bands were finally visualized using an enhanced chemiluminescence (ECL) detection kit according to the manufacturer’s instructions.

### Multiple alignment analysis

All ALV sequences were retrieved from the National Center for Biotechnology Information Genbank database for comparative analysis. The deduced amino acid sequences were aligned using the MegAlign software (DNASTAR, USA). Protein structures were visualised using SPDBV (Version 4.10).

### Structural modeling and prediction

Three-dimensional structures of ALV MA, CA, RT, and IN were generated using homology modeling via SWISS-MODEL (templates: PDB 5KZ9.1 for MA, 98.06% identity; PDB 5A9E.1 for CA, 96.65% identity; PDB 7SR6.1 for RT, 38.52% identity; and the RSV transfer complex cryo-EM structure for IN, 98.92% identity). Concurrently, three-dimensional structures were predicted using AlphaFold 3, with model quality assessed by pIDDT scores. Acetylation at specific K residues was simulated to evaluate structural changes, including alterations in secondary structure, hydrogen bonding patterns, and nucleic acid-binding groove architecture. Three-dimensional structural representations were generated for visualization, and hydrogen bond interactions were analyzed using PyMOL. Predictions of secondary structure were made using IBS (Version 1.0).

### Statistical analysis

Statistical analysis were performed using GraphPad Prism 6.0 (La Jolla, CA, USA). Comparisons between two groups were analyzed using the unpaired t-test. Comparisons between three or more groups were analyzed using either the one-way or two-way ANOVA test. Results are expressed as means ± SEM. Values of P < 0.05 were considered statistically significant.

## Supporting information

S1 Table MS identified information. (PDF)

S2 Table Primers for site-directed mutagenesis. (PDF)

S3 Table The sequence of siRNAs was used for gene knockdown. (PDF)

S4 Table Primers for RT-qPCR. (PDF)

S1 Fig MS/MS spectrum of the acetylated peptide for MA13. (PDF)

S2 Fig MS/MS spectrum of the acetylated peptide for MA67. (PDF)

S3 Fig MS/MS spectrum of the acetylated peptide for MA72. (PDF)

S4 Fig MS/MS spectrum of the acetylated peptide for CA197. (PDF)

S5 Fig MS/MS spectrum of the acetylated peptide for RT13. (PDF)

S6 Fig MS/MS spectrum of the acetylated peptide for IN21. (PDF)

S7 Fig MS/MS spectrum of the acetylated peptide for IN119. (PDF)

S8 Fig MS/MS spectrum of the acetylated peptide for IN129. (PDF)

S9 Fig MS/MS spectrum of the acetylated peptide for IN178. (PDF)

S10 Fig MS/MS spectrum of the acetylated peptide for IN211. (PDF)

S11 Fig MS/MS spectrum of the acetylated peptide for IN250. (PDF)

S12 Fig MS/MS spectrum of the acetylated peptide for IN256. (PDF)

S13 Fig Location of KAc sites in MA. (A) Homology modeling of MA using PDB 5KZ9.1 as a template. (B) Three-dimensional structure of MA predicted by AlphaFold 3. (C) Three-dimensional structure of MA with simulated acetylation at K13, K67, and K72, as predicted by AlphaFold 3. Predicted hydrogen bonds (green dashed lines) between the target residue and adjacent amino acids are highlighted in the magnified view.
(PDF)

S14 Fig Location of KAc sites in CA. (A) Homology modeling of CA using PDB 5A9E.1 as a template. (B) Three-dimensional structure of CA predicted by AlphaFold 3. (C) Three-dimensional structure of CA with simulated acetylation at K196, as predicted by AlphaFold 3. Predicted hydrogen bonds (green dashed lines) between the target residue and adjacent amino acids are highlighted in the magnified view.
(PDF)

S15 Fig Location of KAc sites in RT. (A) Homology modeling of RT using PDB 7SR6.1 as a template. (B) Three-dimensional structure of RT predicted by AlphaFold 3. (C) Three-dimensional structure of RT with simulated acetylation at K13, as predicted by AlphaFold 3. Predicted hydrogen bonds (green dashed lines) between the target residue and adjacent amino acids are highlighted in the magnified view.
(PDF)

S16 Fig Location of KAc sites in IN. (A) Homology modeling of IN using PDB 8E14.1 as a template. (B) Three-dimensional structure of IN predicted by AlphaFold 3. (C) Three-dimensional structure of IN with simulated acetylation at K21, K119, K129, K178, K211, K250, and K256, as predicted by AlphaFold 3. Predicted hydrogen bonds (green dashed lines) between the target residue and adjacent amino acids are highlighted in the magnified view.
(PDF)

S17 Fig HAT1 knockdown does not induce significant cytotoxicity or apoptosis. (A) Cell viability assessed by CCK-8 assay. DF-1 cells were transfected with HAT1 siRNA (50 nM), NC siRNA (50 nM), or mock control (transfection reagent alone). At 24, 48, and 72 h post-transfection, cell viability was measured and normalized to the control group. Data are presented as mean ± SEM from three independent experiments. (B) Quantitative analysis of apoptosis rates. Cells were harvested at 48 h post-transfection, stained with Annexin V/PI, and analyzed by flow cytometry. Percentages of early apoptotic (Annexin V ⁺ /PI⁻) and late apoptotic (Annexin V ⁺ /PI⁺) cells are shown. (C–E) Representative flow cytometry plots and corresponding phase-contrast micrographs of cells in the HAT1 siRNA group (C), NC siRNA group (D), and mock control group (E).
(PDF)
