# Supplementary material for: Acetylation-mediated regulation of ALV viral proteins: Implications for retroviral inhibition
Source: PLoS Pathog. 2026 May 18;22(5):e1014229. doi: 10.1371/journal.ppat.1014229 (PMC13193608; doi:10.1371/journal.ppat.1014229)
Supplement: S1 Table — (PDF) [file ppat.1014229.s001.pdf]

S1 Table. MS identified information.

KAc positio	Protein accession	Posi tion	Amin o acid	Protein description	Gene name	PEP	Score	Ch arg	Modified sequence	Mass error	MS /M	Intensity
MA13	Q7SQ99	13	K	Gag protein OS=Avian leukosis virus OX=11864	gag	4.46E-22	207.7	2	VISSACK(1)TYCGK	-1.366	1	829010
MA67	Q7SQ99	67	K	OS=Avian leukosis virus OX=11864 GN=gag PE=4	gag	6.21E-05	106	2	AMVLGK(1)SGELK	1.3605	2	634390
MA72	Q7SQ99	72	K	OS=Avian leukosis virus OX=11864 GN=gag PE=4	gag	7.05E-24	128.5	2	SGELK(1)TWGLVLGALK	-0.441	1	69376
CA197	Q7SQ99	435	K	OS=Avian leukosis virus OX=11864 GN=gag PE=4	gag	1.64E-05	116.1	2	QK(1)SQPDIQQLIR	-1.092	1	330790
RT13	Q7SQ98	13	K	Pol polyprotein OS=Avian leukosis virus OX=11864	pol	5.31E-22	134.5	3	WK(1)PDHTPVWIDQWPLPEGK	-0.67	1	183580
IN21	Q7SQ98	593	K	OS=Avian leukosis virus OX=11864 GN=pol PE=3	pol	0.000299	78.16	2	ALSK(1)ACNISMQQAR	-0.768	1	60029
IN119	Q7SQ98	691	K	OS=Avian leukosis virus OX=11864 GN=pol PE=3	pol	0.000619	72.33	2	AIK(1)TDNGSCFTSK	0.4883	1	48074
IN129	Q7SQ98	701	K	OS=Avian leukosis virus OX=11864 GN=pol PE=3	pol	7.79E-08	107	2	TDNGSCFTSK(1)STR	-2.432	1	42188
IN178	Q7SQ98	750	K	OS=Avian leukosis virus OX=11864 GN=pol PE=3	pol	0.003428	59.65	2	VLAEGDGFMK(1)R	0.691	1	35490
IN211	Q7SQ98	783	K	OS=Avian leukosis virus OX=11864 GN=pol PE=3	pol	0.003635	113.7	2	TPIQK(1)HWR	-0.114	1	105440
IN250	Q7SQ98	822	K	OS=Avian leukosis virus OX=11864 GN=pol PE=3	pol	0.007923	69.09	2	GYAAVK(1)NR	0.5742	1	26414
IN256	Q7SQ98	828	K	OS=Avian leukosis virus OX=11864 GN=pol PE=3	pol	0.014868	52.84	2	DTDK(1)VIWVPSR	3.7536	1	21540
