# Supplementary material for: Acetylation-mediated regulation of ALV viral proteins: Implications for retroviral inhibition
Source: PLoS Pathog. 2026 May 18;22(5):e1014229. doi: 10.1371/journal.ppat.1014229 (PMC13193608; doi:10.1371/journal.ppat.1014229)
Supplement: S2 Table — (PDF) [file ppat.1014229.s002.pdf]

S19 Table . Primers for site-directed mutagenesis

Gene	Mutation	Primer	Sequence（5'-3'）
MA	K13Q	Forward	CCGCGTGTCAAACTTATTGCGGGAAAACCTCT
		Reverse	ATAAGTTTGACACGCGGACGAAATCACCTTTA
MA	K13R	Forward	CCGCGTGTAGAACTTATTGCGGGAAAACCTCTC
		Reverse	ATAAGTTCTACACGCGGACGAAATCACCTTTA
MA	K67Q	Forward	TACTTGGGCAATCGGGAGAGTTAAAAACCTGG
		Reverse	TCCCGATTGCCCAAGTACCATAGCCCGCTGGG
MA	K67R	Forward	TACTTGGGAGATCGGGAGAGTTAAAAACCTGGG
		Reverse	TCCCGATCTCCCAAGTACCATAGCCCGCTGGG
MA	K72Q	Forward	GAGAGTTACAAACCTGGGGATTGGTTTTGGGG
		Reverse	CCAGGTTTGTAACTCTCCCGATTTCCCAAGTA
MA	K72R	Forward	AGAGTTAAGAACCTGGGGATTGGTTTTGGGGG
		Reverse	CCCAGGTTCTTAACTCTCCCGATTTCCCAAGT
CA	K197Q	Forward	TTAGGCAGCAGTCACAGCCAGATATCCAGCAG
		Reverse	CTGTGACTGCTGCCTAAAGCAGTCAATGATCAC
CA	K197R	Forward	TTAGGCAGAGGTCACAGCCAGATATCCAGCAGC
		Reverse	CTGTGACCTCTGCCTAAAGCAGTCAATGATCA
RT	K13Q	Forward	TCAAATGGCAGCCAGACCACACGCCTGTGTGG
		Reverse	GTCTGGCTGCCATTTGAGCGGGATAGCCAGAT
RT	K13R	Forward	TCAAATGGAGGCCAGACCACACGCCTGTGTGG
		Reverse	GTCTGGCCTCCATTTGAGCGGGATAGCCAGAT

IN	K21Q	Forward	GCTATCCCAAGCGTGTAATATATCTATGCAGCAG
		Reverse	TACACGCTTGGGATAGCGCGCGGGGTCCAATA
IN	K21R	Forward	GCTATCCAGAGCGTGTAATATATCTATGCAGCAGG
		Reverse	TACACGCTCTGGATAGCGCGCGGGGTCCAATA
IN	K119Q	Forward	CCAAAGGCCATACAAACAGATAACGGGTCCTGCTTC
		Reverse	GTTTGTATGGCCTTTGGTCTTCCCAAAACGGC
IN	K119R	Forward	CCAAAGGCCATAAGAACAGATAACGGGTCCTGCTTC
		Reverse	GTTCTTATGGCCTTTGGTCTTCCCAAAACGGC
IN	K129Q	Forward	CCTGCTTCACGTCTCAATCCACGCGAGAG
		Reverse	CTCTCGCGTGGATTGAGACGTGAAGCAGG
IN	K129R	Forward	CTGCTTCACGTCTAGATCCACGCGAGAGTG
		Reverse	CACTCTCGCGTGGATCTAGACGTGAAGCAG1
IN	K178Q	Forward	GGACGGTTTCATGCAAAGAATCCCCACC
		Reverse	GGTGGGGATTCTTTGCATGAAACCGTCC
IN	K178R	Forward	GGACGGTTTCATGAGAAGAATCCCCACC
		Reverse	GGTGGGGATTCTTCTCATGAAACCGTCC
IN	K211Q	Forward	CGATACAACAACACTGGAGACCTACCGTTCTTAC
		Reverse	CCAGTGTTGTTGTATCGGTGTTTTTGTGTTTTCA
IN	K211R	Forward	CGATACAAAGACACTGGAGACCTACCGTTCTTACA
		Reverse	CCAGTGTCTTTGTATCGGTGTTTTTGTGTTTTC
IN	K250Q	Forward	GGACACTGATCAGGTTATTTGGGTACCCTCTCGA
		Reverse	TAACCTGATCAGTGTCCCTGTTTTTCACAGCG

IN	K250R	Forward	GAGGTTATGCCGCTGTGAGAAACAGGGACACTGATAAG
		Reverse	CTTATCAGTGTCCCTGTTTCTCACAGCGGCATAACCTC
IN	K256Q	Forward	GGACACTGATCAGGTTATTTGGGTACCCTCTCGA
		Reverse	TAACCTGATCAGTGTCCCTGTTTTTCACAGCG
IN	K256R	Forward	GGACACTGATAGGGTTATTTGGGTACCCTCTCGA
		Reverse	TAACCCTATCAGTGTCCCTGTTTTTCACAGCG
