# Supplementary material for: Acetylation-mediated regulation of ALV viral proteins: Implications for retroviral inhibition
Source: PLoS Pathog. 2026 May 18;22(5):e1014229. doi: 10.1371/journal.ppat.1014229 (PMC13193608; doi:10.1371/journal.ppat.1014229)
Supplement: S3 Table — (PDF) [file ppat.1014229.s003.pdf]

S20 Table . The sequence of siRNA was used for gene knockdown

| Target Gene | Sense Strand (5′→3′) | Antisense Strand (3′→5′) |
|---|---|---|
| KAT1 | GGCUACAAGGGACUGAAGATT | UCUUCAGUCCCUUGUAGCCTT |
| KAT2A | GAGGAGAUCUAUGGUGAGATT | UCUCACCAUAGAUCUCCUCTT |
| KAT2B | GACAAGCUUCCUCAAGAGATT | UCUCUUGAGGAAGCUUGUCTT |
| KAT3A | GCCAACAGCAGGGAUGAAUTT | AUUCAUCCCUGCUGUUGGCTT |
| KAT3B | GCAGAGGGCGACCAAAUAUTT | AUAUUUGGUCGCCCUCUGCTT |
| KAT4 | GCUGCCAUCAAGCAUGACUTT | AGUCAUGCUUGAUGGCAGCTT |
| KAT5 | GCUGCUCAUCGAGLUCAGUTT | ACUGAACUCGAUGAGCAGCTT |
| KAT6A | GUGCUGUGAUCCUCCUCUUTT | AAGAGGAGGAUCACAGCACTT |
| KAT6B | GAGALCCAGACGUGGUACUTT | AGUACCACGUCUGGAUCUCTT |
| KAT7 | CCCCACUGUAUCACAAUCUTT | AGAUUGUGAUACAGUGGGCTT |
| KAT8 | GGAAACACGAUGAGAUCAATT | UUGAUCUCAUCGUGUUUCCTT |
| KAT9 | GCGUCACAGCGUGGAUAAATT | UUUAUCCACGCUGUGACCCTT |
| KAT13A | GGCGAUCCUCAACCAGUUUTT | AAACUGGUUGAGGAUCGCCTT |
| KAT13B | GAGGAGAUGAGUGGAGACUTT | AGUCUCCACUCAUCUCCUCTT |
| KAT13C | GCAUGAGGGAGAAAUAUCUTT | AGAUAUUUCUCCCUCAUGCTT |
| KAT13D | GAAUUCCAGGCCAGAGUUUTT | AAACUCUGGCCUGGAAUUCTT |
| HDAC1 | GCGCUGUGAAGCUGAACAATT | UUGUUCAGCUUCACAGCGCTT |
| HDAC2 | GCUUGCCAUCCUUGAGUUATT | UAACUCAAGGAUGGCAAGCTT |
| HDAC3 | GCGGAGAGUGGUCGUUAUUTT | AAUAACGACCACUCUCCGCTT |
| HDAC4 | GUCCCAGCUCUCCAAACAATT | UUGUUUGGAGAGCUGGGACTT |

| | | |
|---|---|---|
| HDAC7 | GGGUUCUGCUUCUUCAACUTT | AGUUGAAGAAGCAGAACCCTT |
| HDAC8 | GGAUGGCAAGUGCAAGAGATT | UCUCUUGCACUUGCCAUCCTT |
| HDAC9 | GCCAGCUGCCAAUGAGUUUTT | AAACUCAUUGGCAGCUGGCTT |
| HDAC10 | GCACUGAGACAUCCUCUUUTT | AAAGAGGAUGUCUCAGUGCTT |
| HDAC11 | GAUAUCACACUGGCUAUUATI | UAAUAGCCAGUGUGAUAUCTT |
| SIRT1 | GAUGAACCACUUGCUAUCATT | UGAUAGCAAGUGGUUCAUCTT |
| SIRT2 | GAAGCCAGACAUCGUAUUUTT | AAAUACGAUGUCUGGCUUCTT |
| SIRT3 | GCCUGACAUCGUGUUCUUUTT | AAAGAACACGAUGUCAGGCTT |
| SIRT4 | AACGUGGAUGCGCUUCACATT | UGUGAAGCGCAUCCACGUUTT |
| SIRT5 | GCGAGGUCAUGCUGAGUAATT | UUACUCAGCAUGACCUCGCTT |
| SIRT6 | GCAGAGGGAAGCUAAGAGATT | UCUCUUAGCUUCCCUCUGCTT |
| SIRT7 | GGGAAUGCAAACGCACUCATT | UGAGUGCGUUUGCAUUCCCTT |
