# Supplementary material for: Acetylation-mediated regulation of ALV viral proteins: Implications for retroviral inhibition
Source: PLoS Pathog. 2026 May 18;22(5):e1014229. doi: 10.1371/journal.ppat.1014229 (PMC13193608; doi:10.1371/journal.ppat.1014229)
Supplement: S4 Table — (PDF) [file ppat.1014229.s004.pdf]

S21 Table . Primers for RT-qPCR

Target Gene	Sense Strand (5′→3′)	Antisense Strand (3′→5′)
Gp85	CGCCGCAATTACACAGAG	ACGTGTCCACAACGGTAGC
β-actin	ATGAAGCCCAGAGCAAAAGA	GGGGTGTTGAAGGTCTCAAA
KAT1	CGCTGGTAACCTGTCAACTCTCTTC	ATCATCTGCCTCCACACAGTCAAAC
KAT2A	TGACATCCAATGAGCAAGTGAAGGG	ATCGGCATAGGTGAGGAAGTAGAGG
KAT2B	TTAGCCACTTGCCCTCAAAGGAAAG	AAGGTGTCTCCAGGTGCCAGTAG
KAT3A	GACCCTTCCCAGCCTCAAACAAC	GCATCTTCCGACCACACTCCTTG
KAT3B	TGATGAGAGCAACAACCAGCAGAC	CAGGATGGCAGCGAGCAGTTG
KAT4	CCGAGGAGGAGGAGGAAGAAGAAG	TCATCCGAGTCCAGGTCACTGTC
KAT5	GTGACCCGAAGCGTAAAGTGGAG	GACGGATGCCTGCGAGGTTTC
KAT6A	GCCATTCTCCTGCTGTGACTTCC	TGCTTGTGGTGTGCCTGCTAAC
KAT6B	CGACCACAGCAGCCCAGTTTC	GAGATGGCACAGAAATGGCACTTTG
KAT7	GAGGAATTCGGGACTAAGCAAGGAG	CATACTCGCTGGTCAGGTTCTCAAG
KAT8	GGCACTCAGCAGAGGTGATTCAG	TTCGTCCAAGCGGCGGTTAAAG
KAT9	CAAAGTATGGGCTCTCGGCACAG	ACATCACAGCCACGACAGCAATC
KAT13A	GACATCTCATCCAGCAGCCAAGG	ATTCACGACGAAGAAGAAGCCATCC
KAT13B	AATACGGATGTCACAAGCCTGGAAG	CCATCATCACTGCGGATTCCTGAC
KAT13C	GCGTGCCATCGGTAGTGAGTG	AGGCGAAGGCTCCATCTGATCC
KAT13D	CAGTAGCGTGTGGAGCGGTAATG	AAGTCTGTGGCTGCTGCTGTTG
HDAC1	CCACAACCTACTGCTGAACTACGG	GGGCGGATGGATCTCAGGAATTTG
HDAC2	TTGAACAGACAGCAGACAGACATGG	TCAGTAACTCAAGGATGGCAAGCAC

HDAC3	ACCGTTTGGGCTGCTTTAACCTC	ATCGTGCCACATTGCGGACTG
HDAC4	GTTGGCACAGGAGCAGGAGTTG	TGGGCATTACGACCGTTCTGAAAG
HDAC7	GCAGGCAGTGTCACCGAGTTG	CCACCGAGTTGAAGAAGCAGAACC
HDAC8	CTGGGAGGAGGAGGCTACAACC	GGACAGCGTTCTGCCAAGGATG
HDAC9	CTCAGATGGCTACAGTTGGCATGG	GGCTGGGTGAGGTAATGTGGAATC
HDAC10	TGCCGTGGAATAAGGTTGGAATGG	CCAGAGGAGACAAGAACCAGTTCAG
HDAC11	ATCCCACCAGTCCTCTTTCTTCCC	GCCCAGCCTCTATCAACAGCAAG
SIRT1	GGGGTGTCTGTGTCTTGTGGAATAC	TTGAGGATCTGGAAGGTCTGGGAAG
SIRT2	TCAAGCAACACCCAGAGCCTTTC	CAGCAGCCGCATGAAGTAGTGG
SIRT3	CTGCCGCCAGTGCTGTTCTAG	CGACGACACTCCTTCTTCCGAATG
SIRT4	TCGGAAGGTGTGGGGCTCTATG	TTGGCTGGTGGGAGGAGAACTG
SIRT5	CCGAACTGGTGCTGGGAAATGG	CGGTAATGATGGCGATATGCTTTGC
SIRT6	AAGCAACCAAGCACGACAGACAG	GAGTCAGCACTTTCCACCACCAC
SIRT7	AACGCTCAGTGTCTTGGCTTGC	CTTGTCTGGAACCACCTCAGTCATC
