# Supplementary figures and images for: Acetylation-mediated regulation of ALV viral proteins: Implications for retroviral inhibition
Source: PLoS Pathog. 2026 May 18;22(5):e1014229. doi: 10.1371/journal.ppat.1014229 (PMC13193608; doi:10.1371/journal.ppat.1014229)

| Raw file                       | Scan number | Mass analyzer | Score  | m/z      | Proteins |
|--------------------------------|-------------|---------------|--------|----------|----------|
| BE397PIAc_ALV_Slot1-24_1_20764 | 12460       | TOF           | 207.67 | 708.3365 | Q7SQ99   |

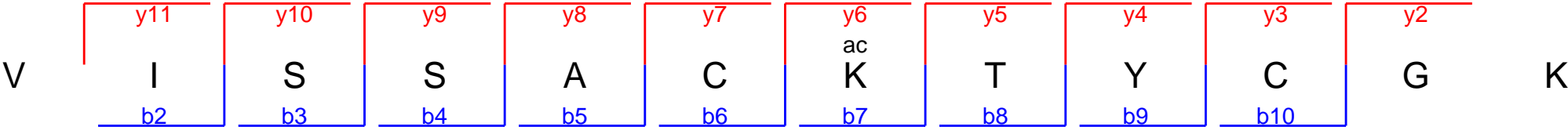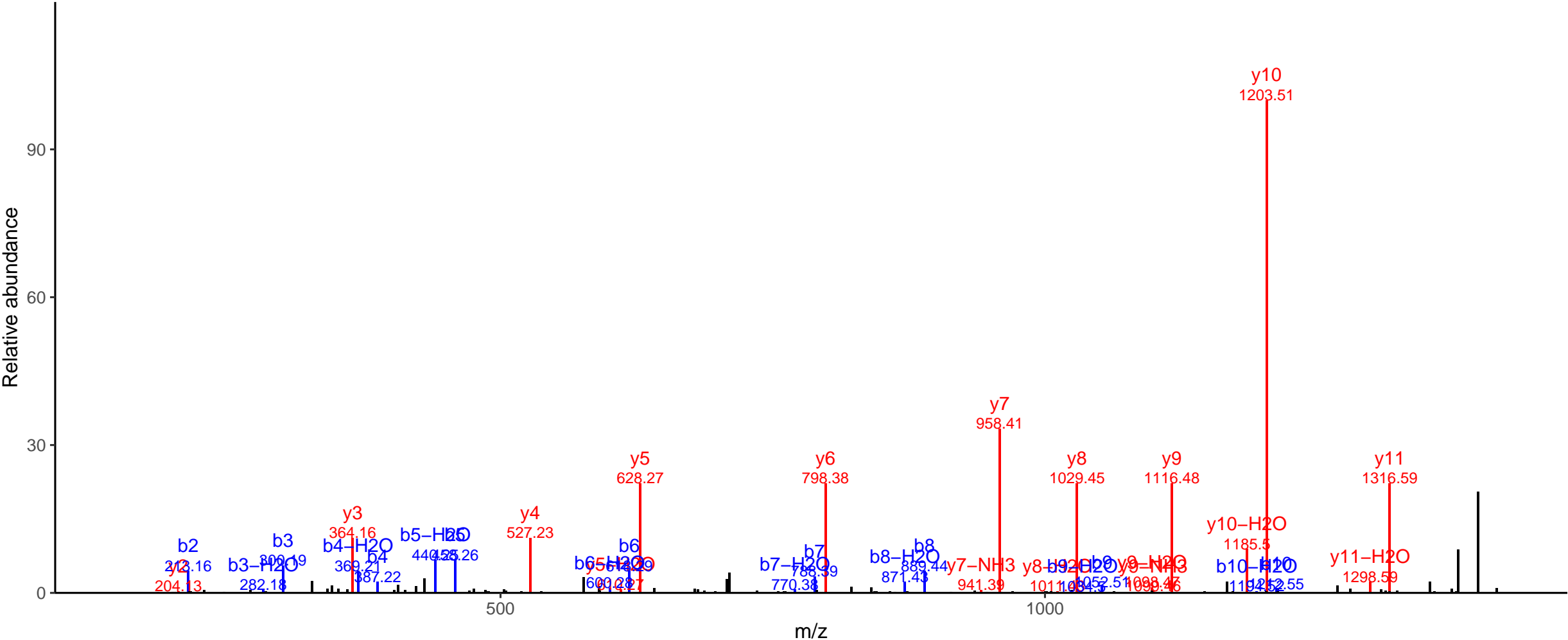

Supplement: S1 Fig — (PDF) [file ppat.1014229.s005.pdf]

| Raw file                       | Scan number | Mass analyzer | Score | m/z      | Proteins |
|--------------------------------|-------------|---------------|-------|----------|----------|
| BE397PIAc_ALV_Slot1-24_1_20764 | 6420        | TOF           | 106   | 587.8287 | Q7SQ99   |

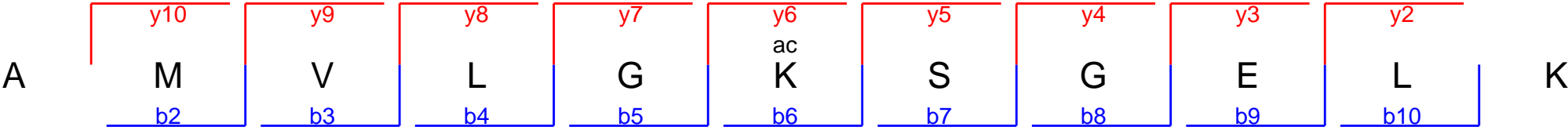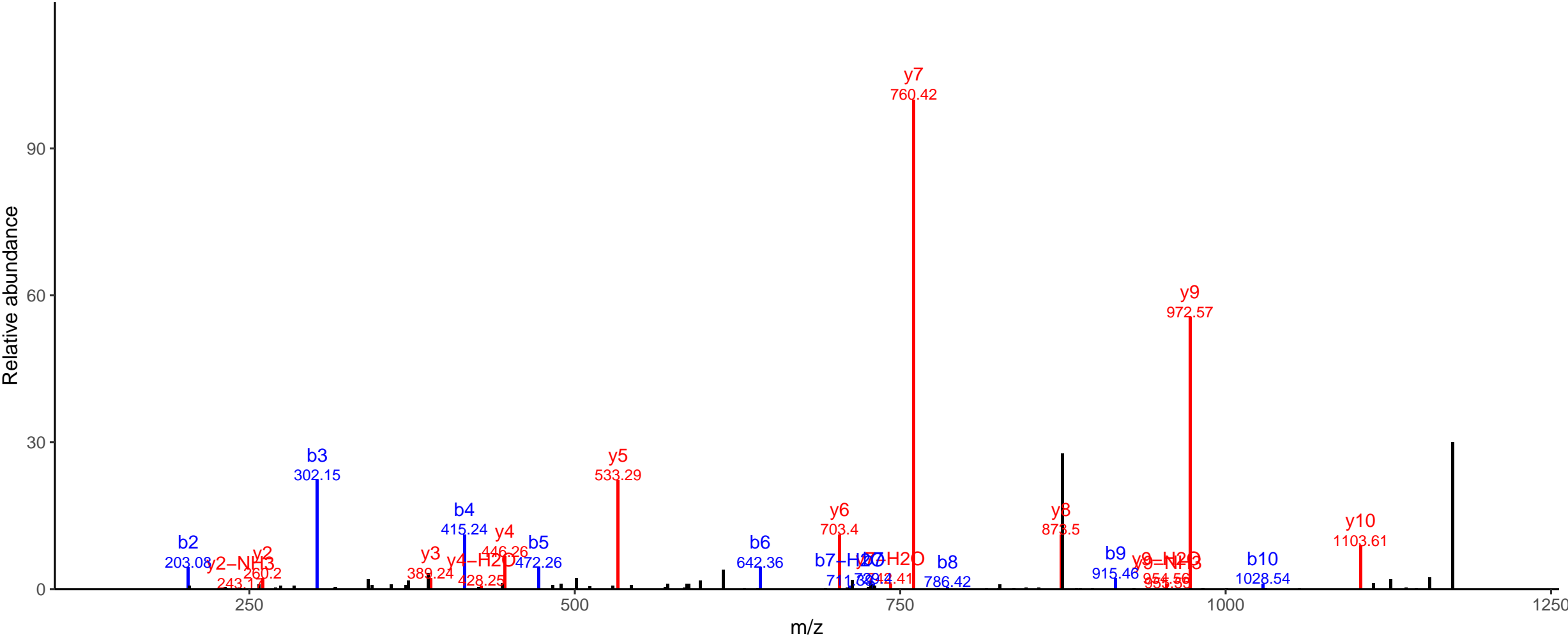

Supplement: S2 Fig — (PDF) [file ppat.1014229.s006.pdf]

| Raw file                       | Scan number | Mass analyzer | Score  | m/z      | Proteins |
|--------------------------------|-------------|---------------|--------|----------|----------|
| BE397PIAc_ALV_Slot1-24_1_20764 | 17880       | TOF           | 128.46 | 807.4667 | Q7SQ99   |

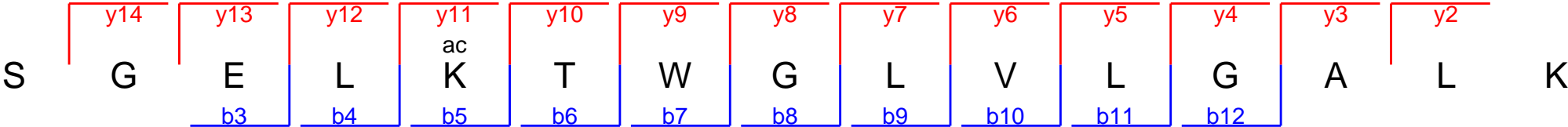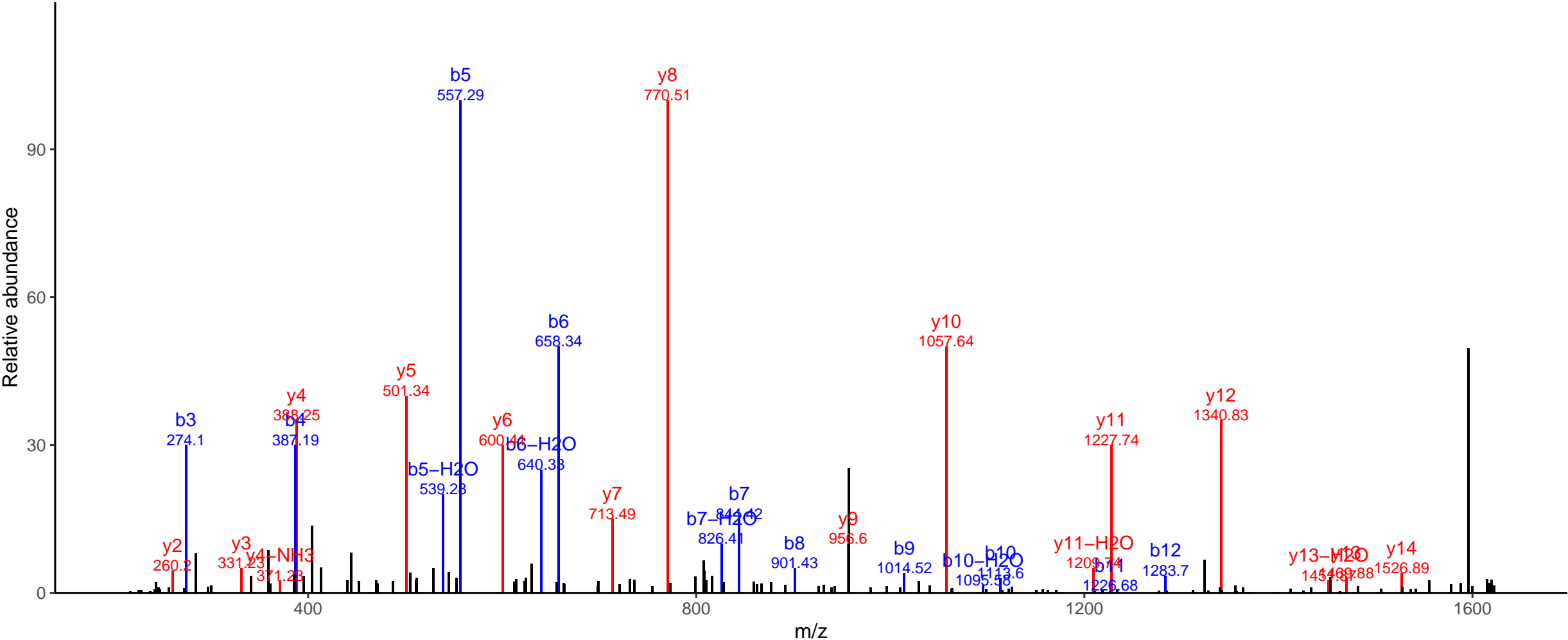

Supplement: S3 Fig — (PDF) [file ppat.1014229.s007.pdf]

| Raw file                       | Scan number | Mass analyzer | Score  | m/z     | Proteins |
|--------------------------------|-------------|---------------|--------|---------|----------|
| BE397PIAc_ALV_Slot1-24_1_20764 | 14511       | TOF           | 116.13 | 748.415 | Q7SQ99   |

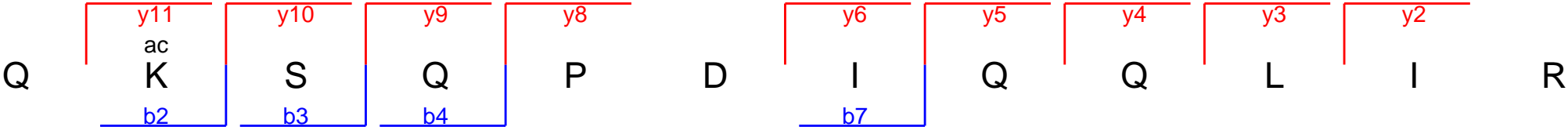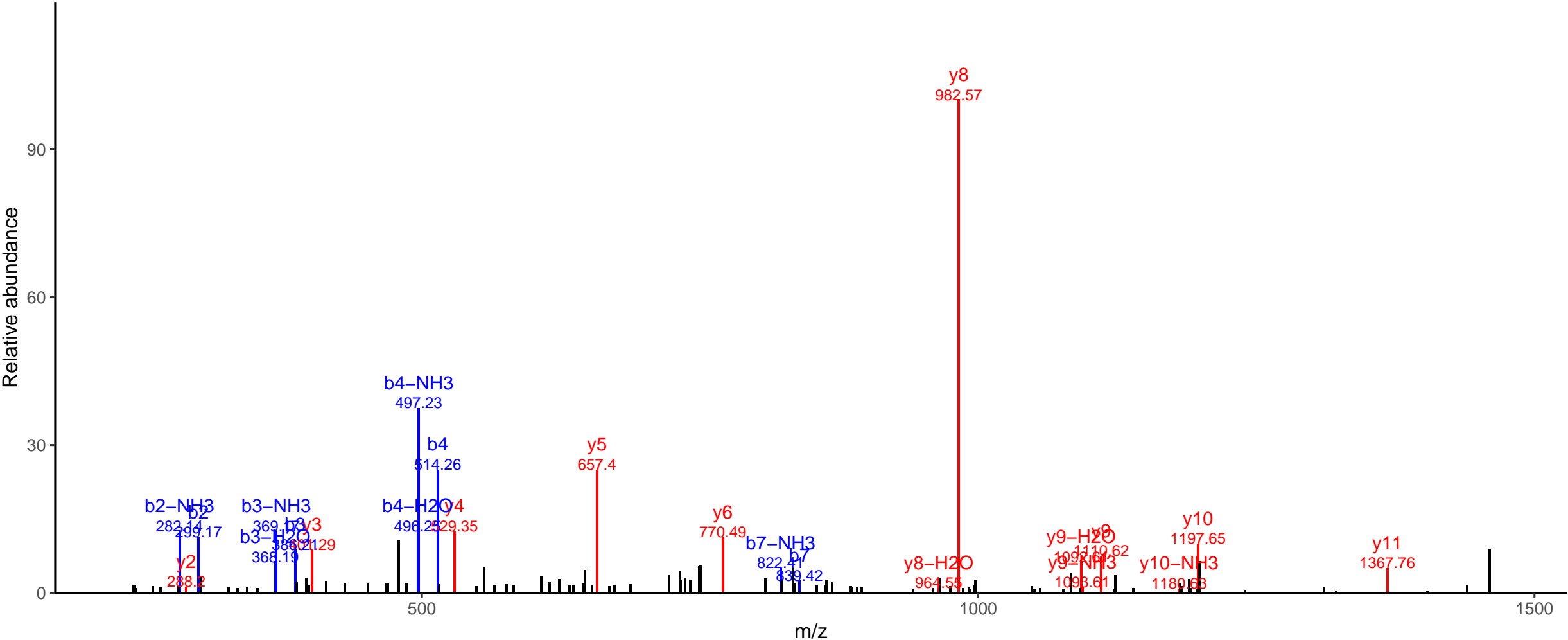

Supplement: S4 Fig — (PDF) [file ppat.1014229.s008.pdf]

| Raw file                       | Scan number | Mass analyzer | Score  | m/z      | Proteins |
|--------------------------------|-------------|---------------|--------|----------|----------|
| BE397PIAc_ALV_Slot1-24_1_20764 | 29775       | TOF           | 134.49 | 791.0672 | Q7SQ98   |

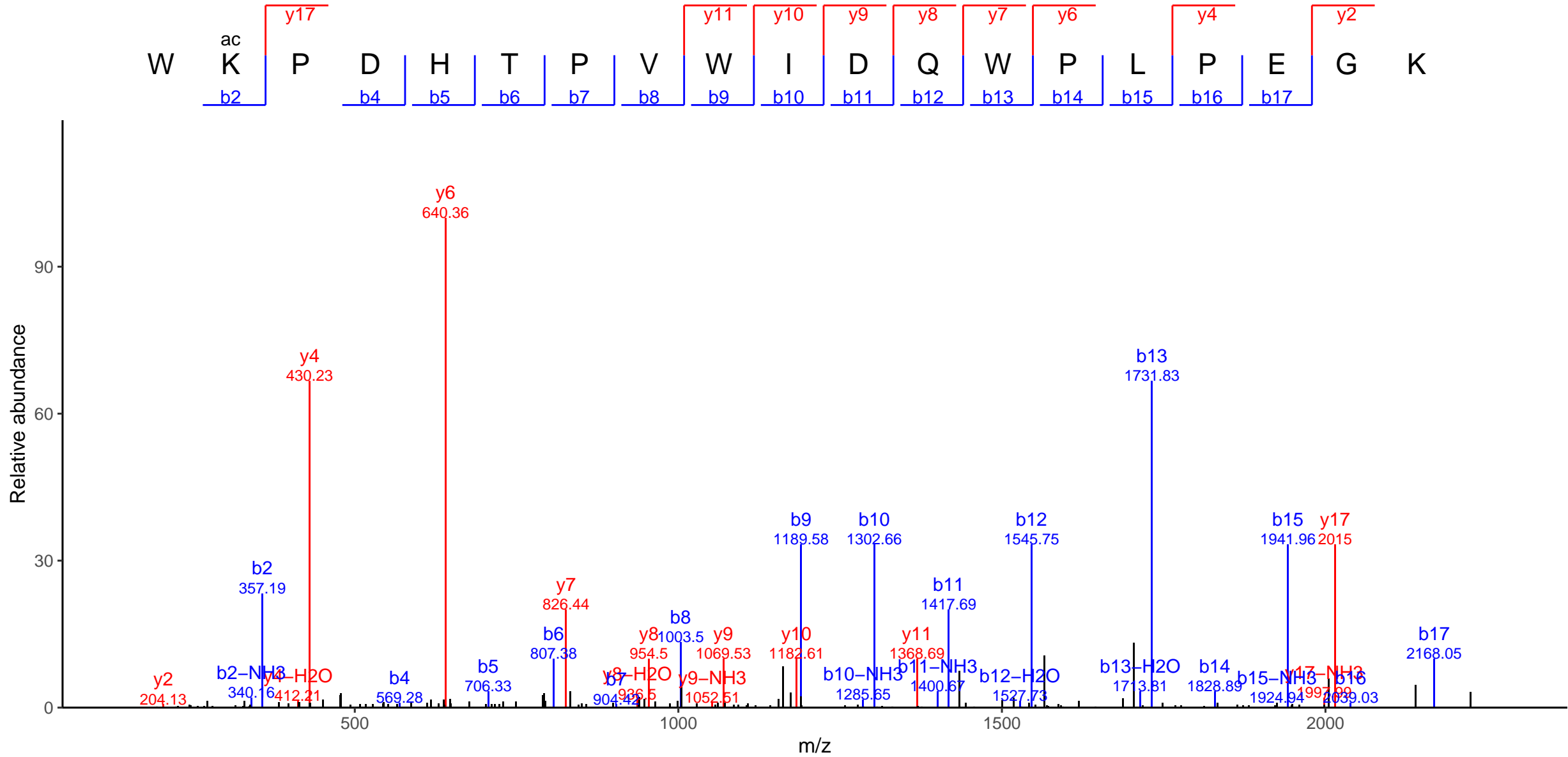

Supplement: S5 Fig — (PDF) [file ppat.1014229.s009.pdf]

| Raw file                       | Scan number | Mass analyzer | Score  | m/z      | Proteins |
|--------------------------------|-------------|---------------|--------|----------|----------|
| BE397PIAc_ALV_Slot1-24_1_20764 | 18041       | TOF           | 78.157 | 810.4032 | Q7SQ98   |

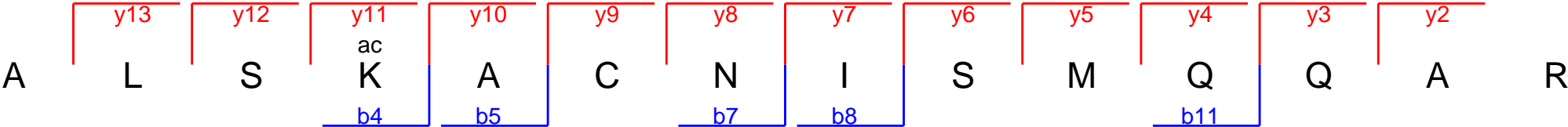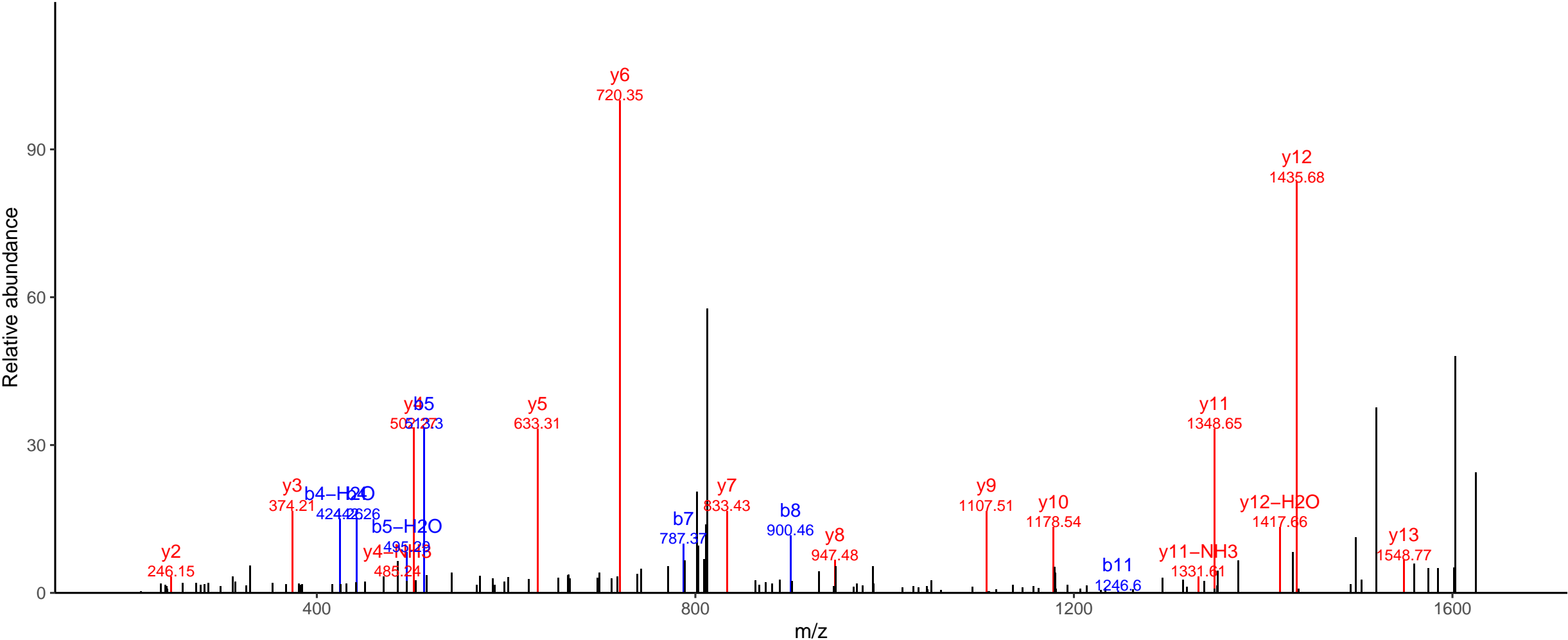

Supplement: S6 Fig — (PDF) [file ppat.1014229.s010.pdf]

| Raw file                       | Scan number | Mass analyzer | Score  | m/z      | Proteins |
|--------------------------------|-------------|---------------|--------|----------|----------|
| BE397PIAc_ALV_Slot1-24_1_20764 | 13862       | TOF           | 72.327 | 735.8483 | Q7SQ98   |

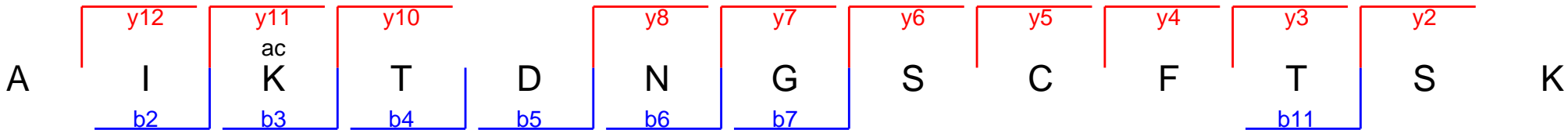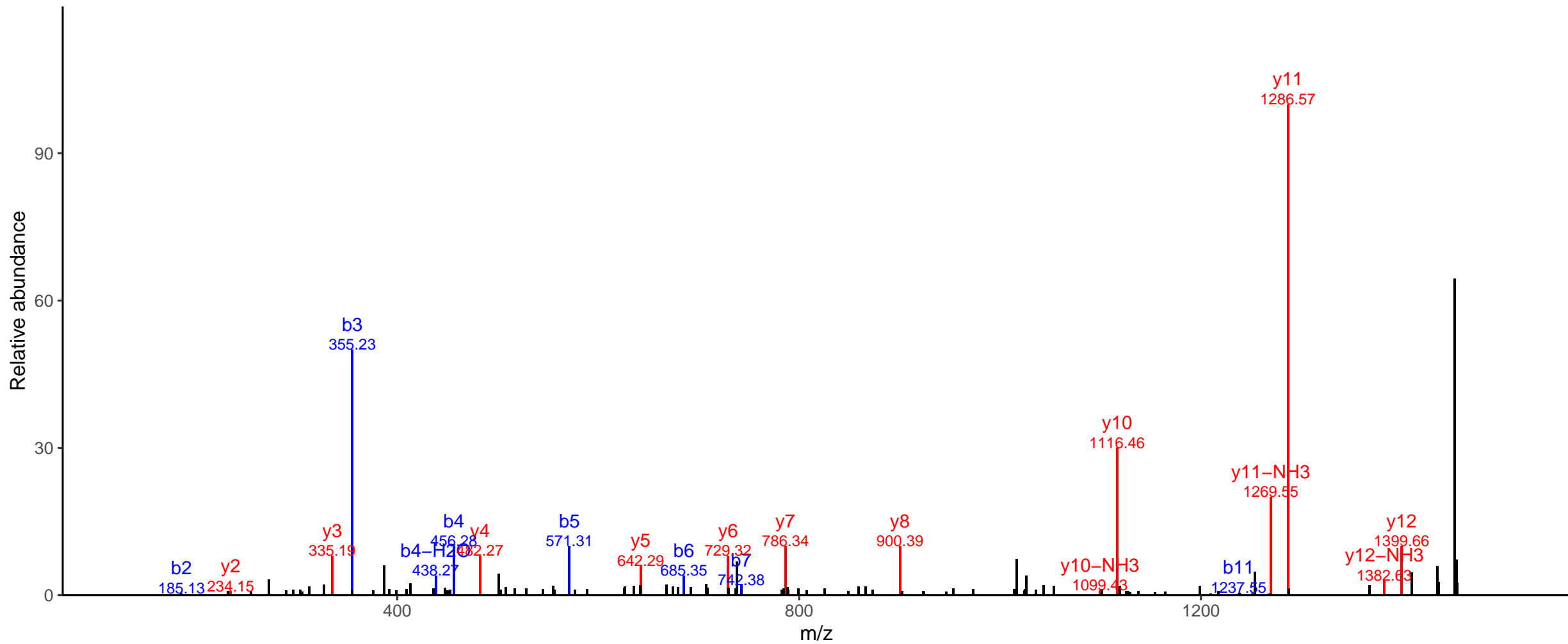

Supplement: S7 Fig — (PDF) [file ppat.1014229.s011.pdf]

| Raw file                       | Scan number | Mass analyzer | Score  | m/z      | Proteins |
|--------------------------------|-------------|---------------|--------|----------|----------|
| BE397PIAc_ALV_Slot1-24_1_20764 | 14644       | TOF           | 106.97 | 751.8306 | Q7SQ98   |

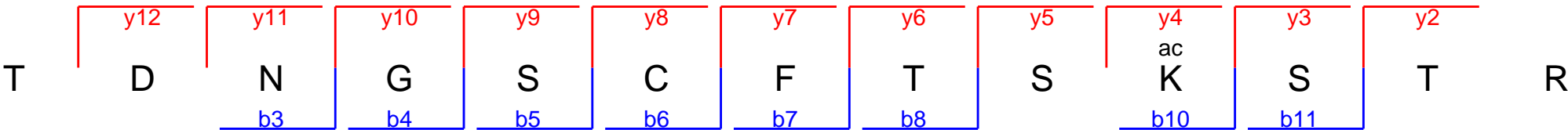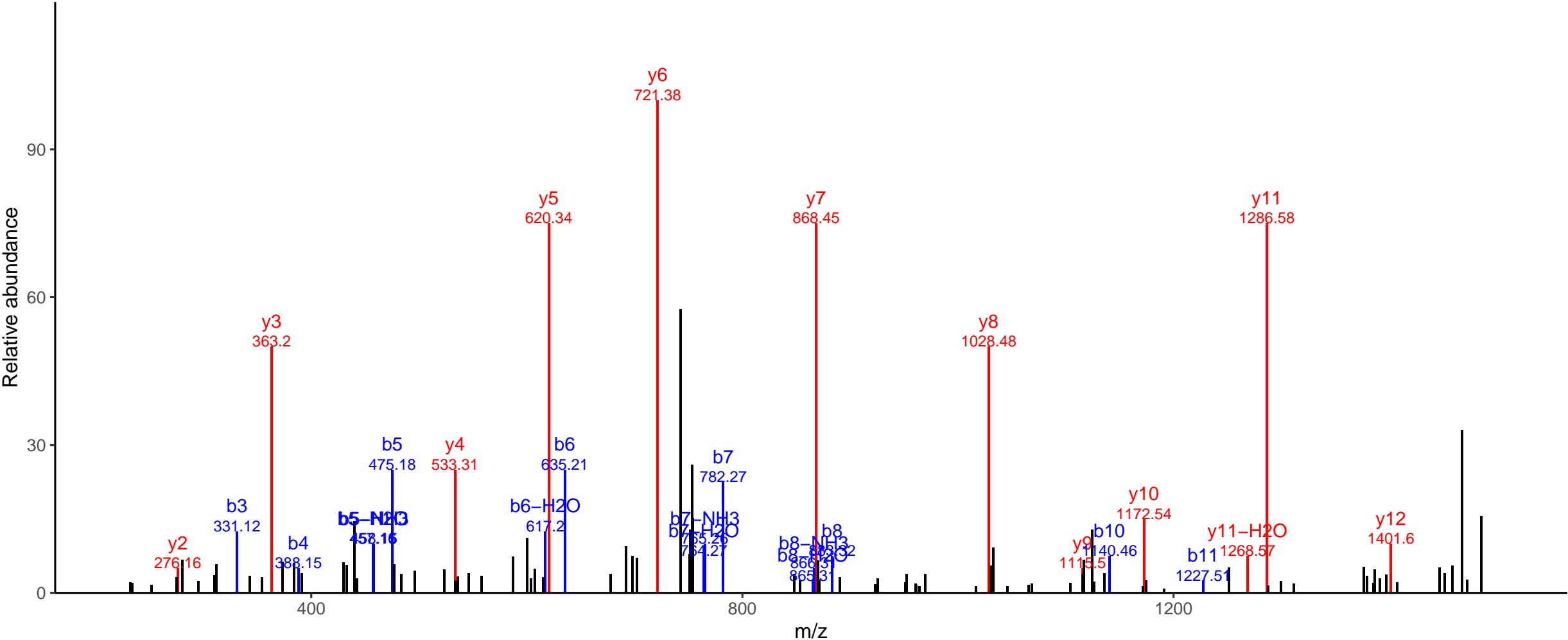

Supplement: S8 Fig — (PDF) [file ppat.1014229.s012.pdf]

| Raw file                       | Scan number | Mass analyzer | Score  | m/z      | Proteins |
|--------------------------------|-------------|---------------|--------|----------|----------|
| BE397PIAc_ALV_Slot1-24_1_20764 | 8465        | TOF           | 59.645 | 632.8214 | Q7SQ98   |

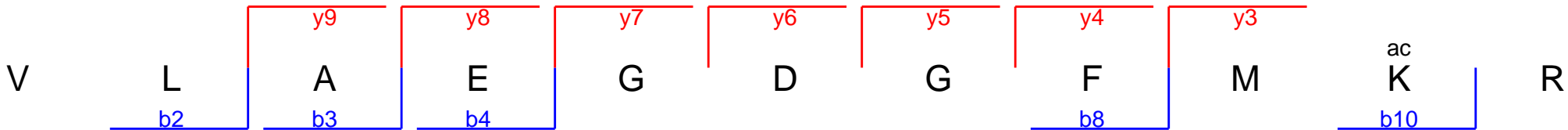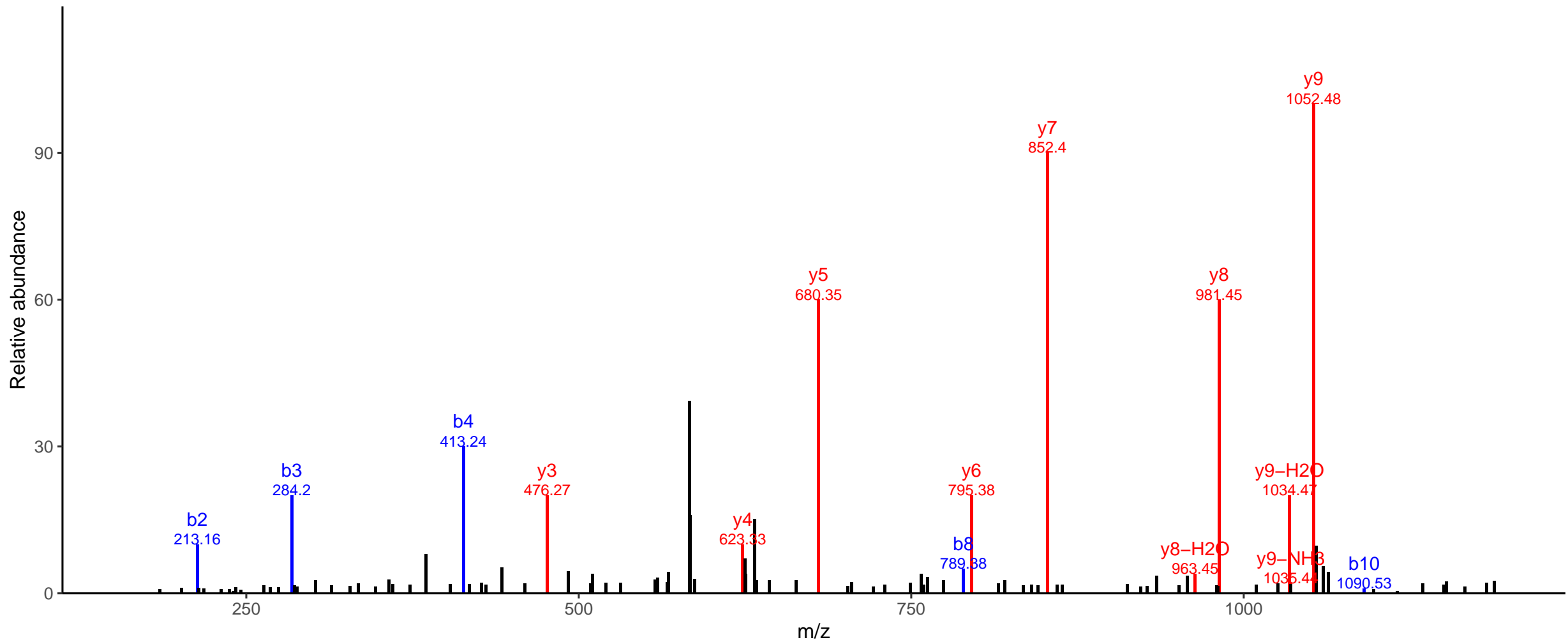

Supplement: S9 Fig — (PDF) [file ppat.1014229.s013.pdf]

| Raw file                       | Scan number | Mass analyzer | Score | m/z      | Proteins |
|--------------------------------|-------------|---------------|-------|----------|----------|
| BE397PIAc_ALV_Slot1-24_1_20764 | 4952        | TOF           | 113.7 | 554.3065 | Q7SQ98   |

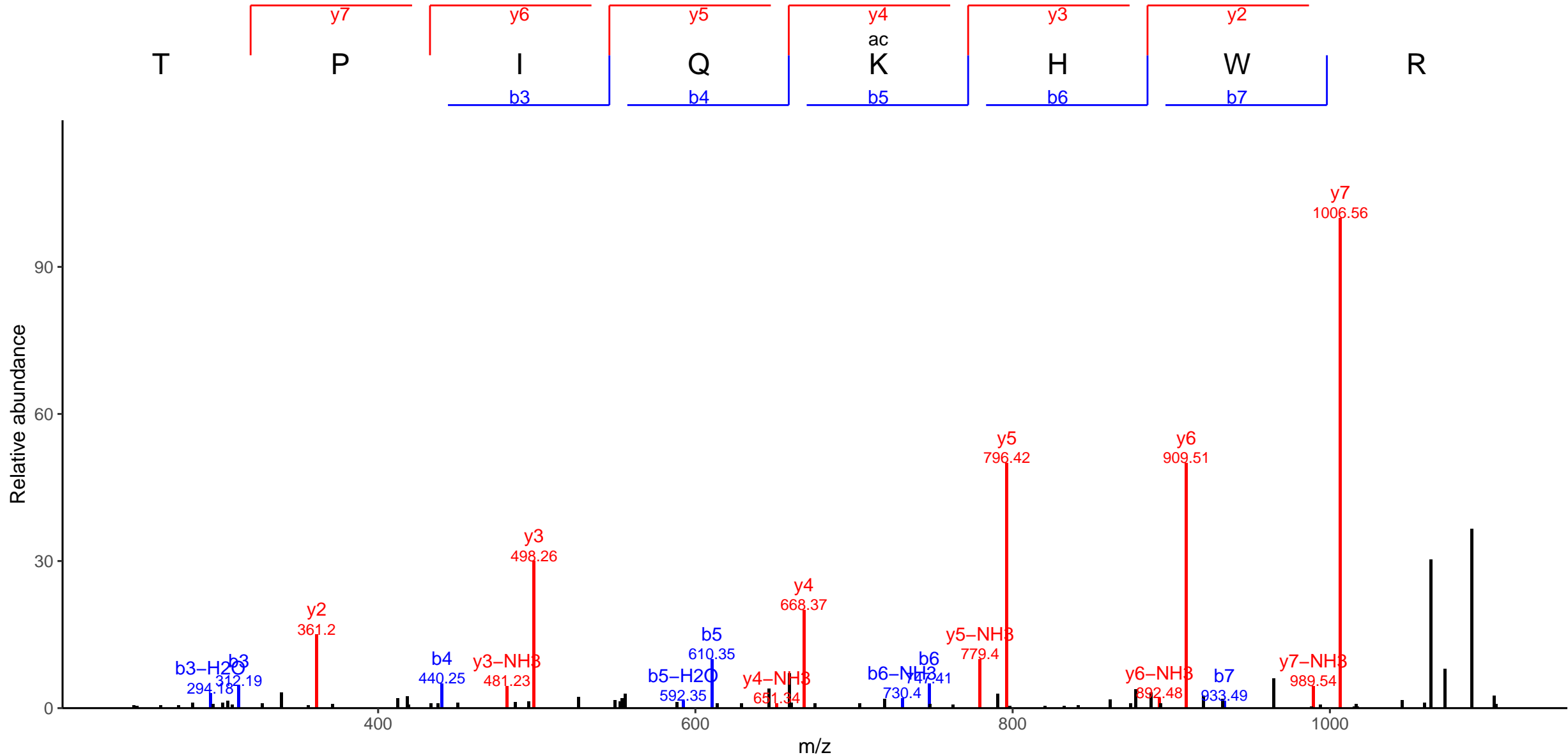

Supplement: S10 Fig — (PDF) [file ppat.1014229.s014.pdf]

| Raw file                       | Scan number | Mass analyzer | Score  | m/z      | Proteins |
|--------------------------------|-------------|---------------|--------|----------|----------|
| BE397PIAc_ALV_Slot1-24_1_20764 | 1992        | TOF           | 69.093 | 460.7511 | Q7SQ98   |

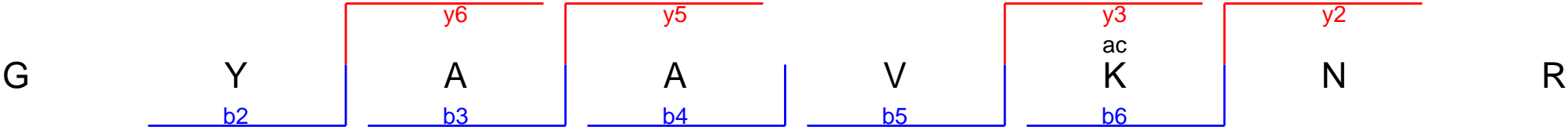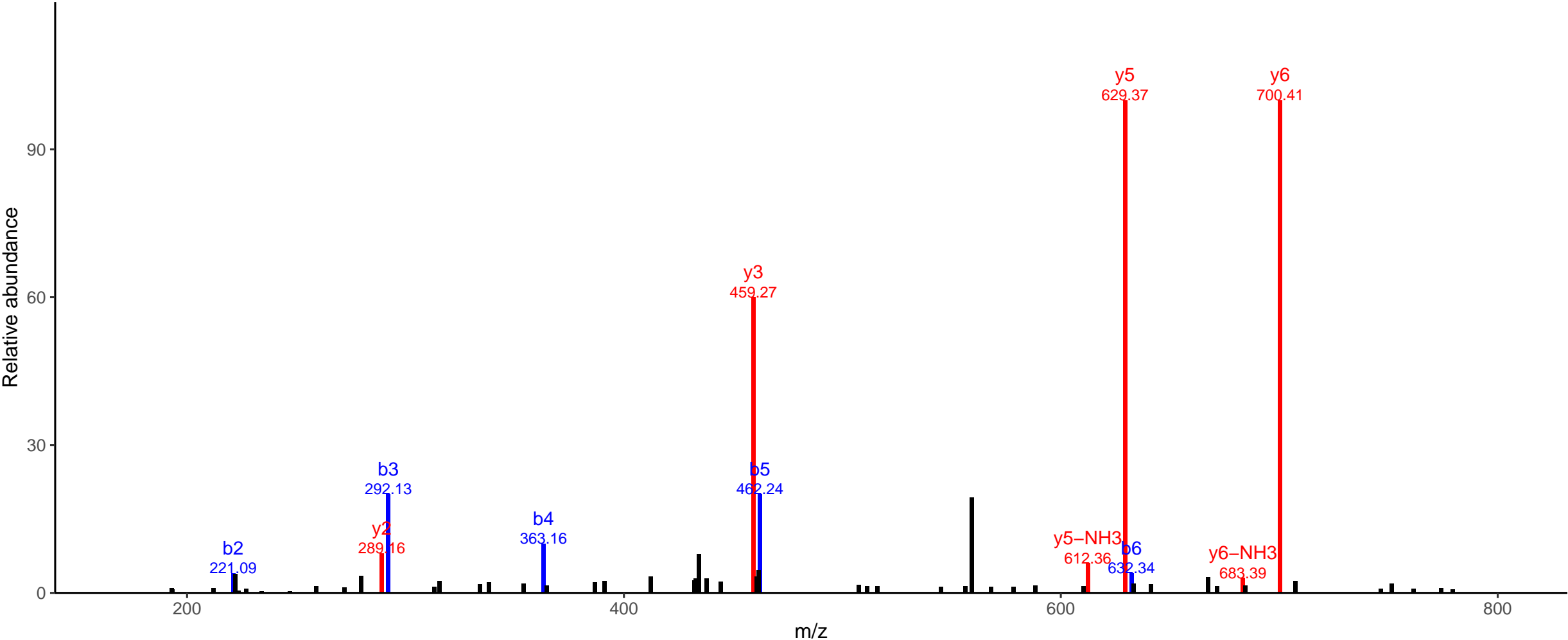

Supplement: S11 Fig — (PDF) [file ppat.1014229.s015.pdf]

| Raw file                       | Scan number | Mass analyzer | Score  | m/z      | Proteins |
|--------------------------------|-------------|---------------|--------|----------|----------|
| BE397PIAc_ALV_Slot1-24_1_20764 | 10978       | TOF           | 52.837 | 679.3592 | Q7SQ98   |

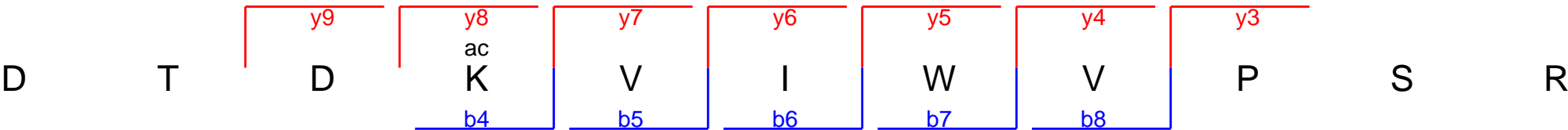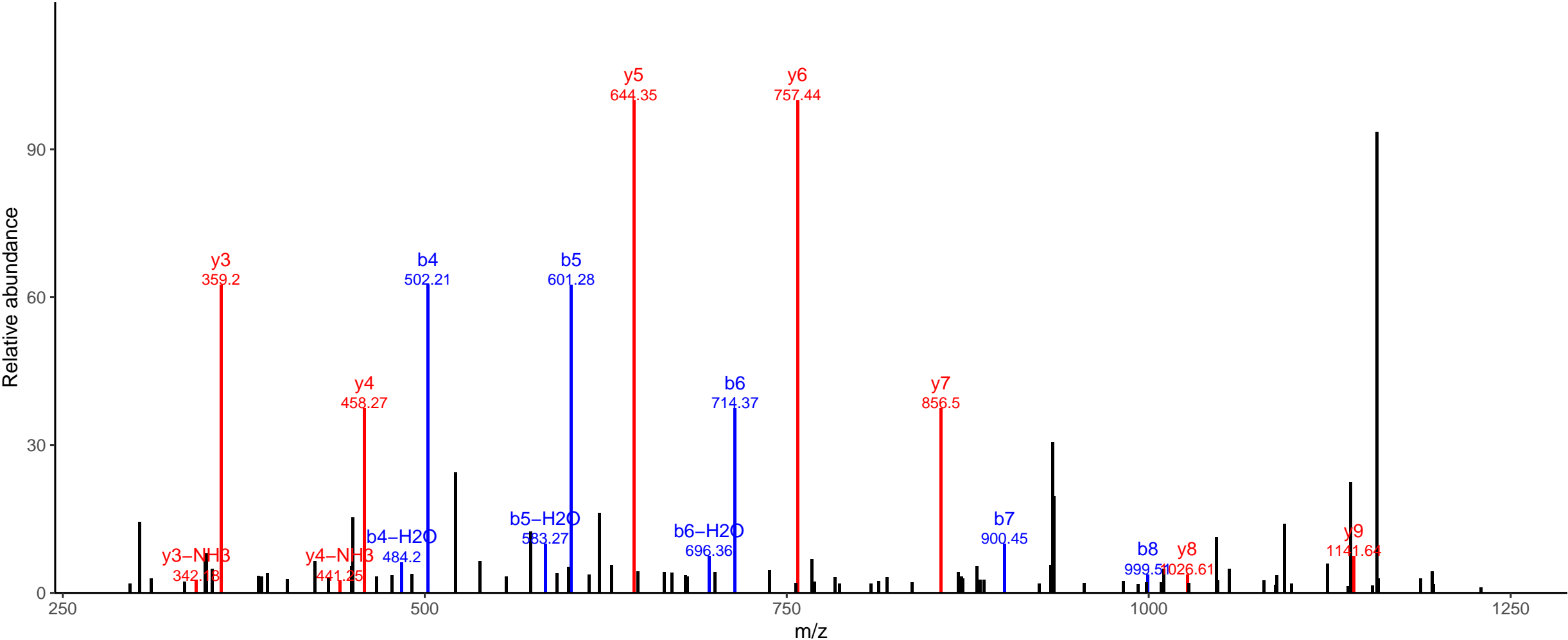

Supplement: S12 Fig — (PDF) [file ppat.1014229.s016.pdf]

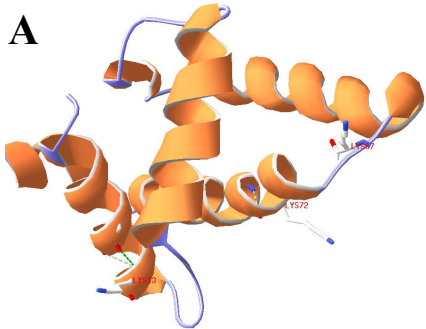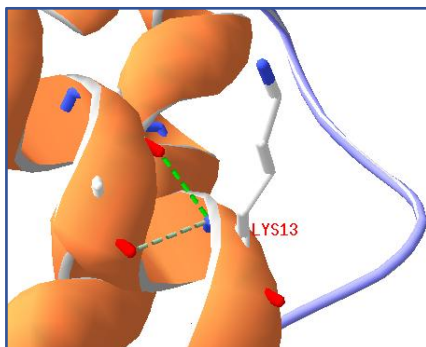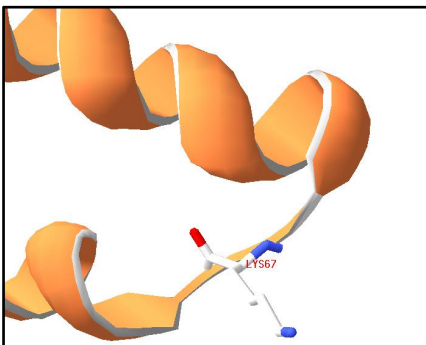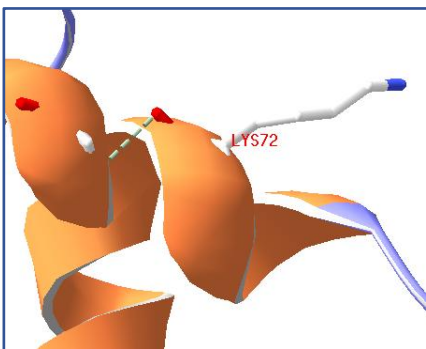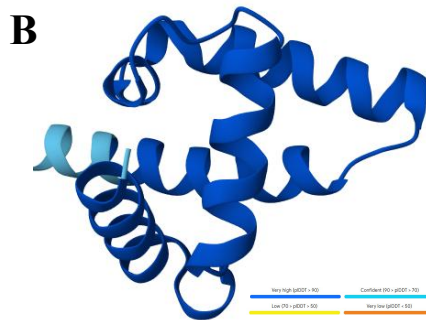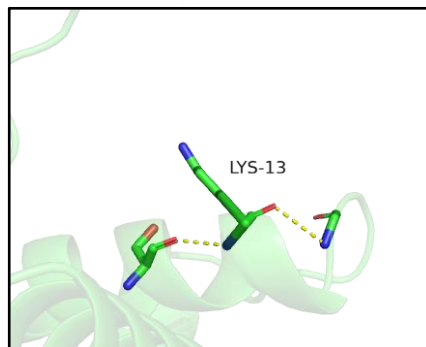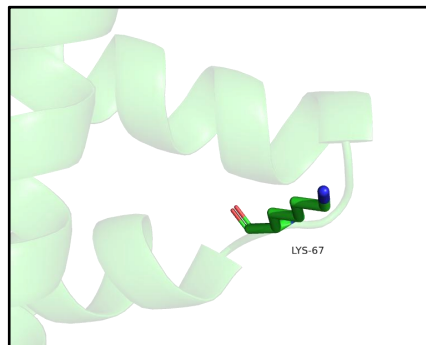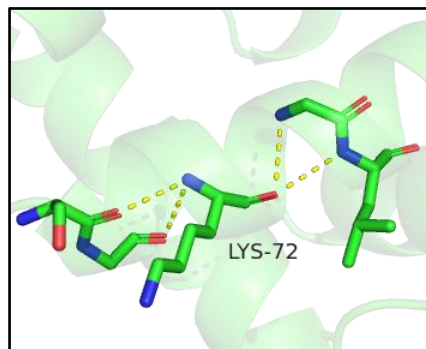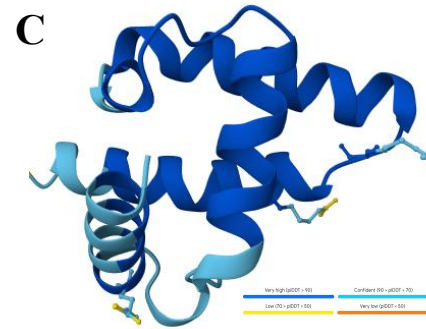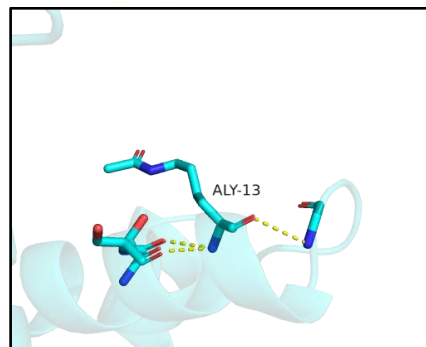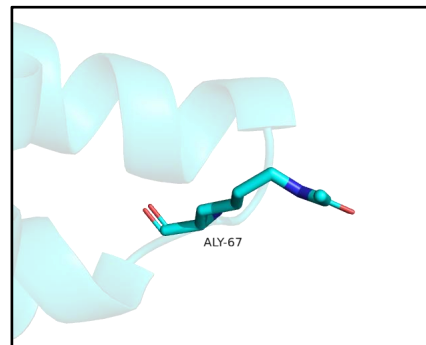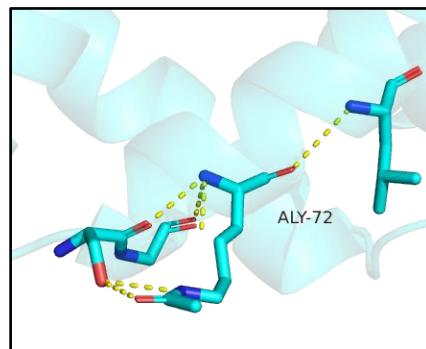

Supplement: S13 Fig — (A) Homology modeling of MA using PDB 5KZ9.1 as a template. (B) Three-dimensional structure of MA predicted by AlphaFold 3. (C) Three-dimensional structure of MA with simulated acetylation at K13, K67, and K72, as predicted by AlphaFold 3. Predicted hydrogen bonds (green dashed lines) between the target residue and adjacent amino acids are highlighted in the magnified view. (PDF) [file ppat.1014229.s017.pdf]

**A**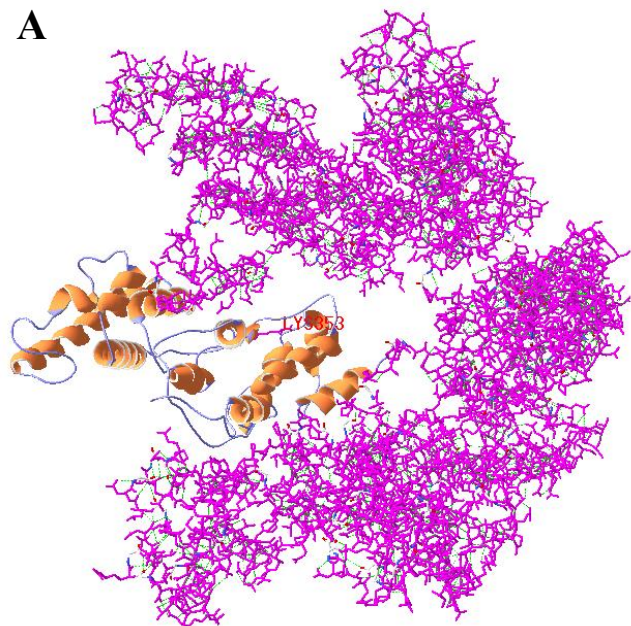**B**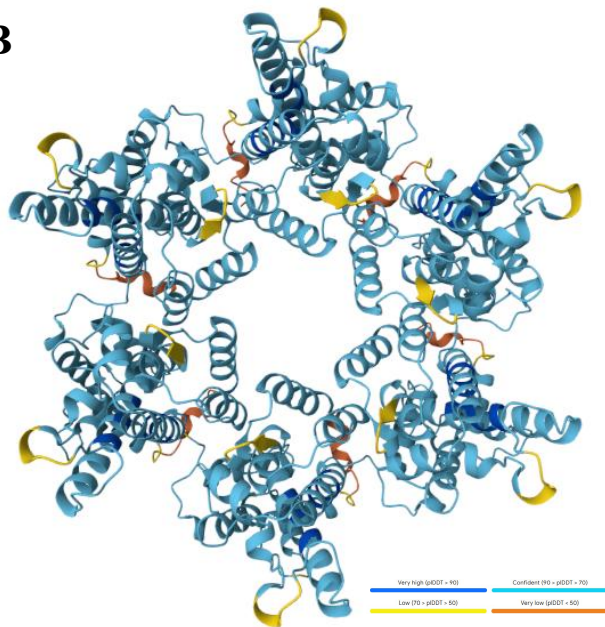**C**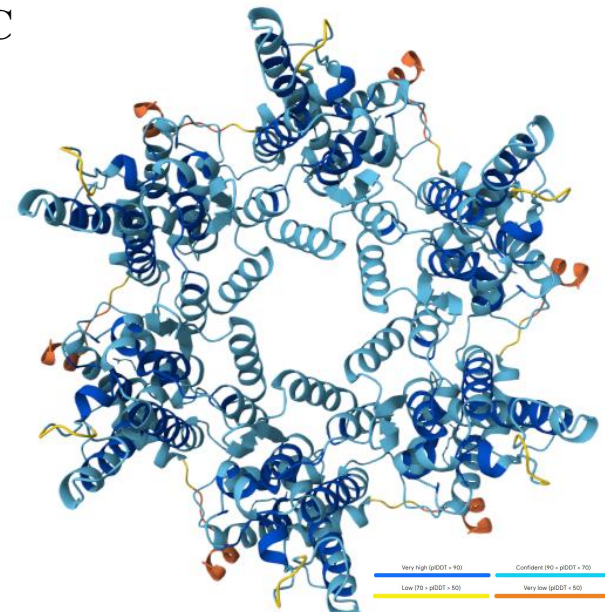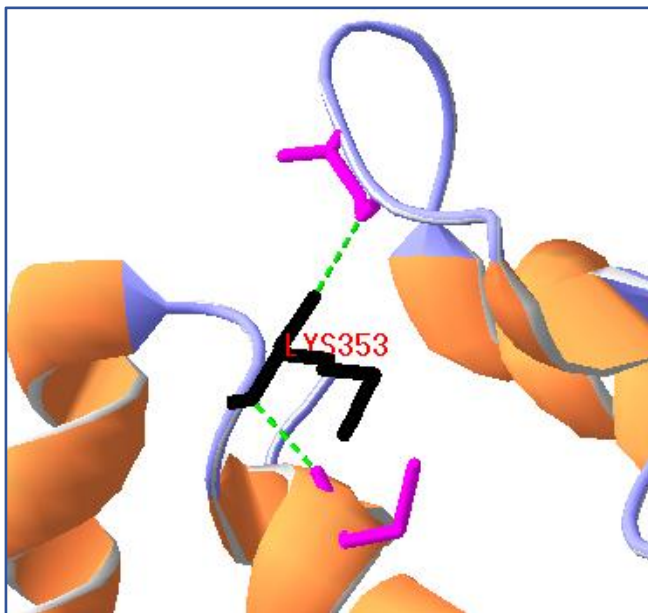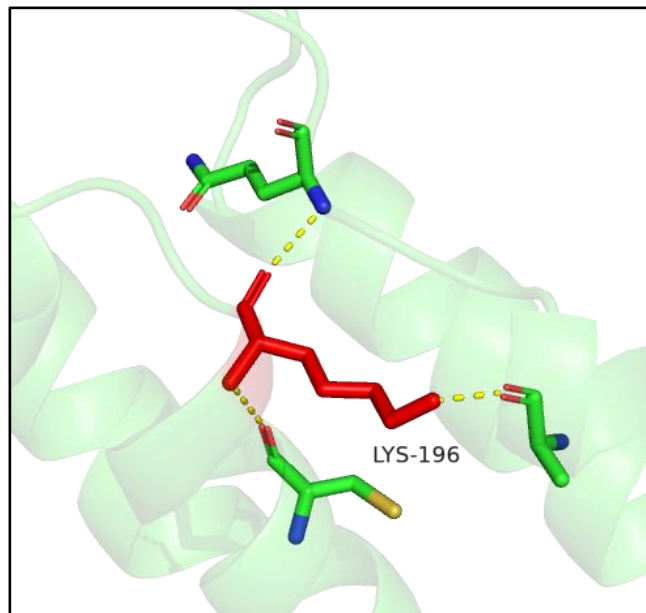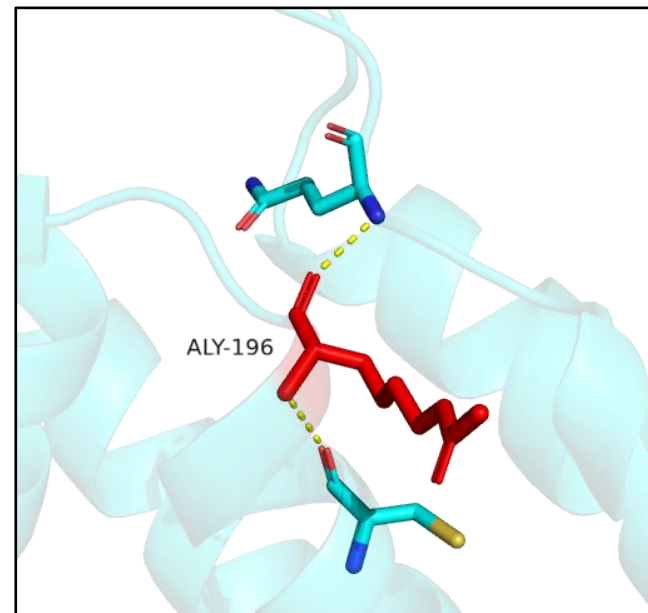

Supplement: S14 Fig — (A) Homology modeling of CA using PDB 5A9E.1 as a template. (B) Three-dimensional structure of CA predicted by AlphaFold 3. (C) Three-dimensional structure of CA with simulated acetylation at K196, as predicted by AlphaFold 3. Predicted hydrogen bonds (green dashed lines) between the target residue and adjacent amino acids are highlighted in the magnified view. (PDF) [file ppat.1014229.s018.pdf]

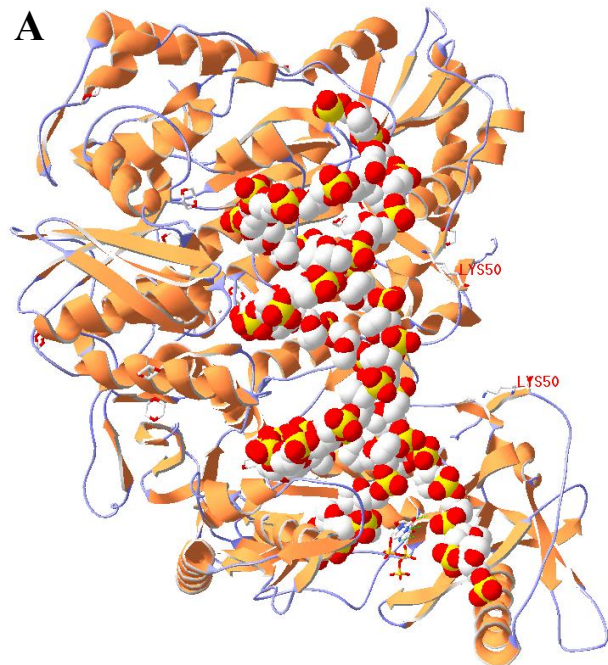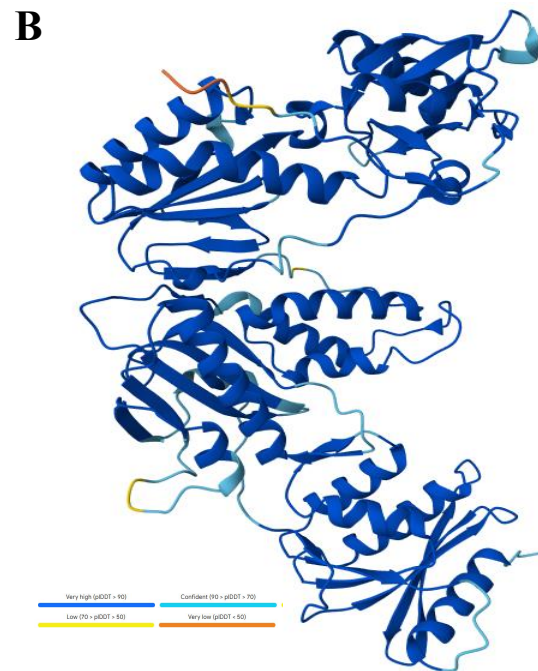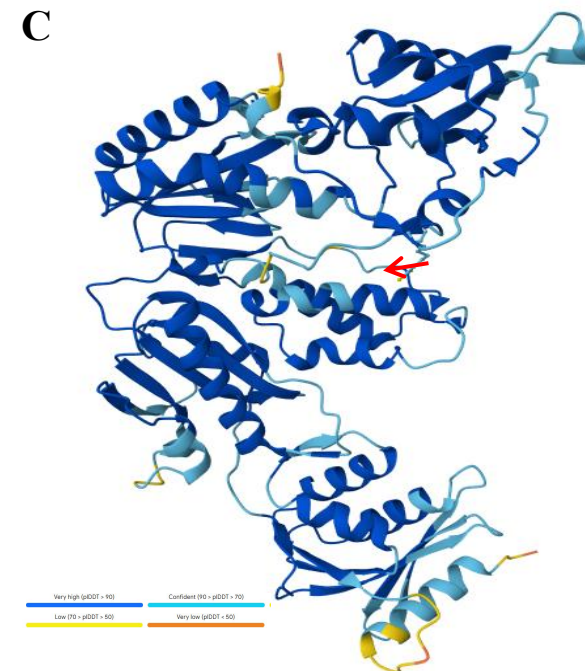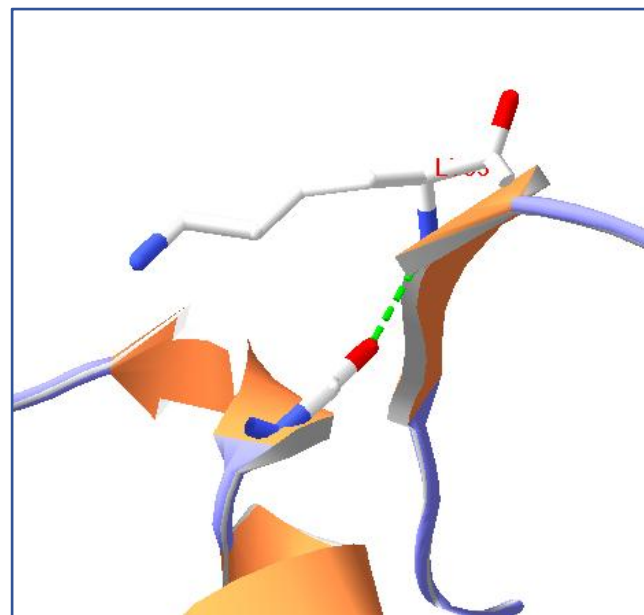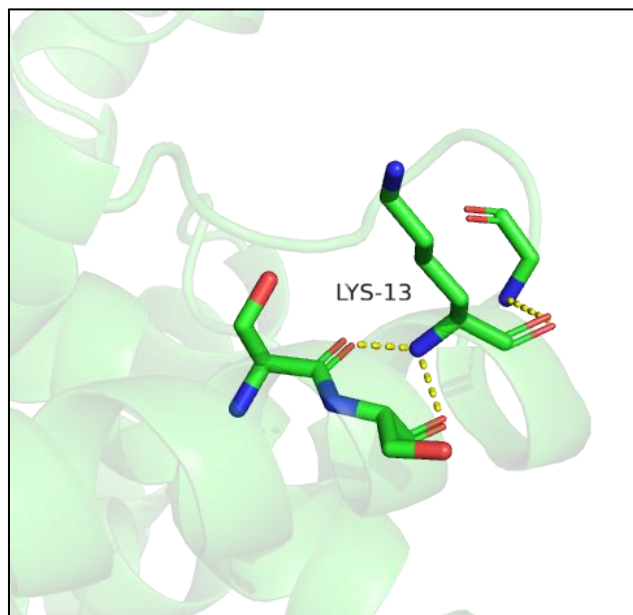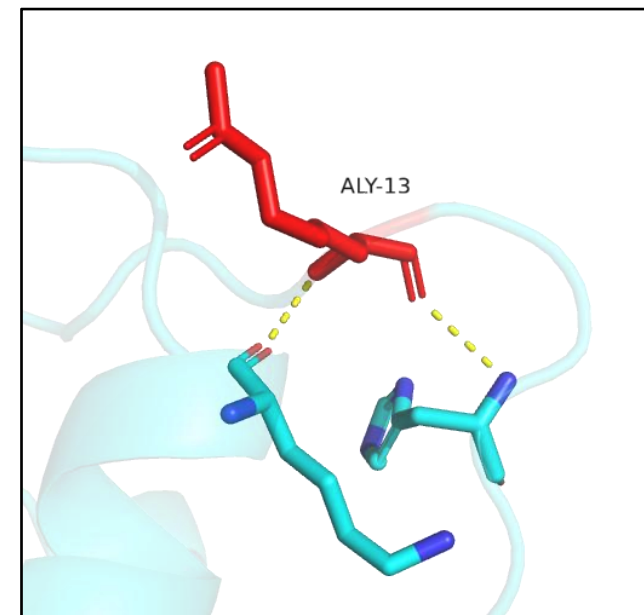

Supplement: S15 Fig — (A) Homology modeling of RT using PDB 7SR6.1 as a template. (B) Three-dimensional structure of RT predicted by AlphaFold 3. (C) Three-dimensional structure of RT with simulated acetylation at K13, as predicted by AlphaFold 3. Predicted hydrogen bonds (green dashed lines) between the target residue and adjacent amino acids are highlighted in the magnified view. (PDF) [file ppat.1014229.s019.pdf]

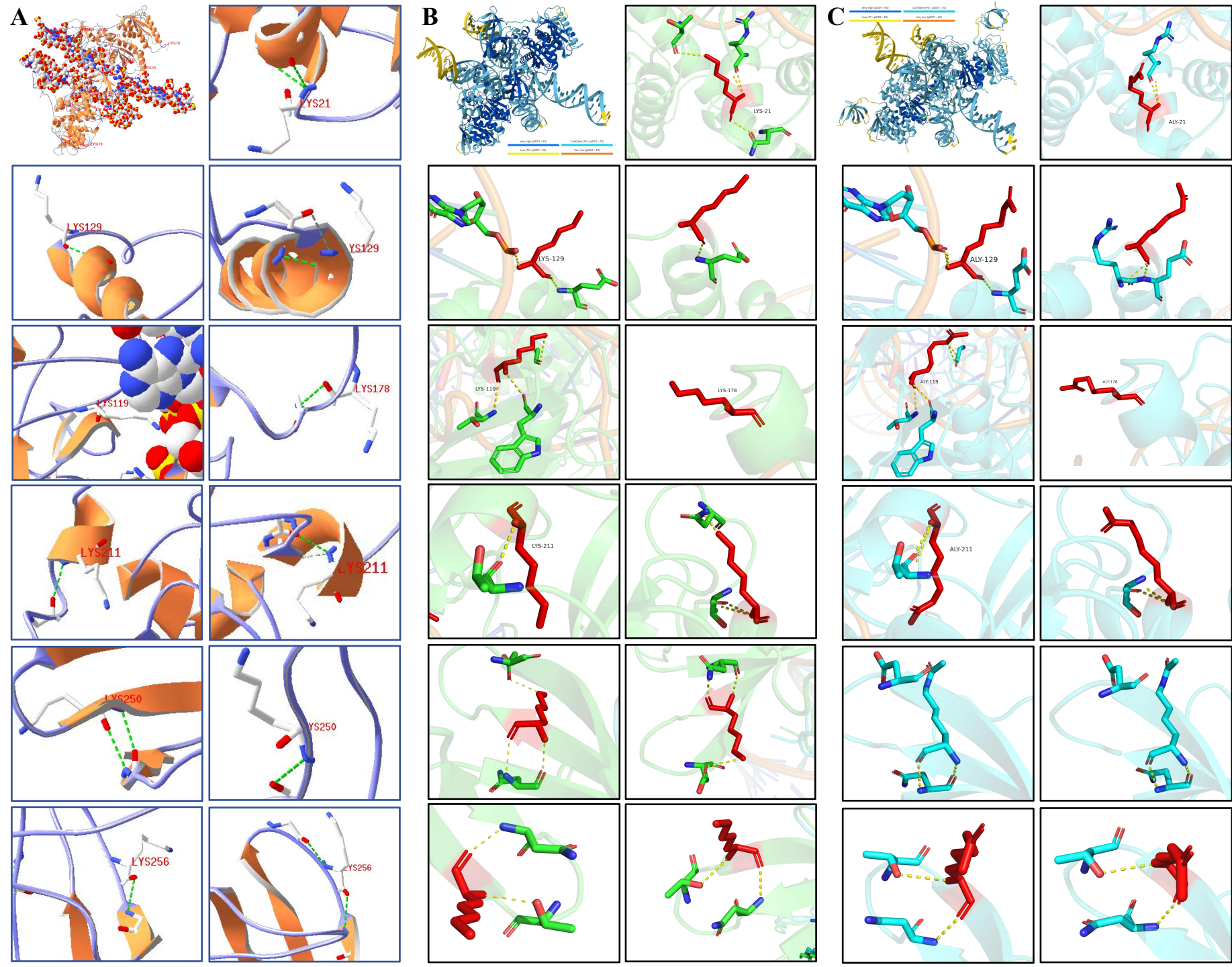

Supplement: S16 Fig — (B) Three-dimensional structure of IN predicted by AlphaFold 3. (C) Three-dimensional structure of IN with simulated acetylation at K21, K119, K129, K178, K211, K250, and K256, as predicted by AlphaFold 3. Predicted hydrogen bonds (green dashed lines) between the target residue and adjacent amino acids are highlighted in the magnified view. (PDF) [file ppat.1014229.s020.pdf]

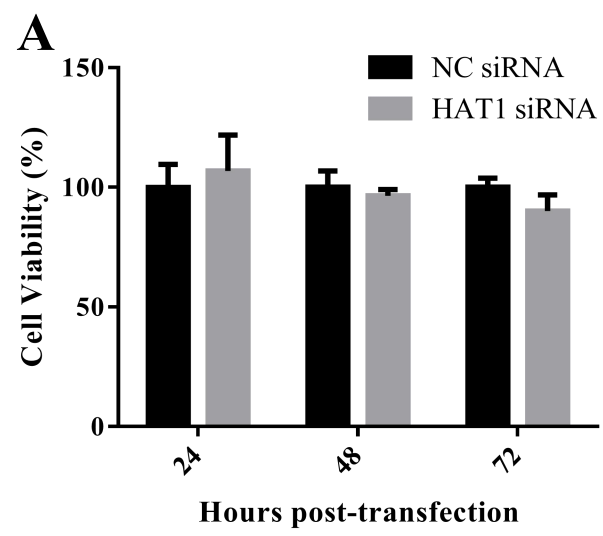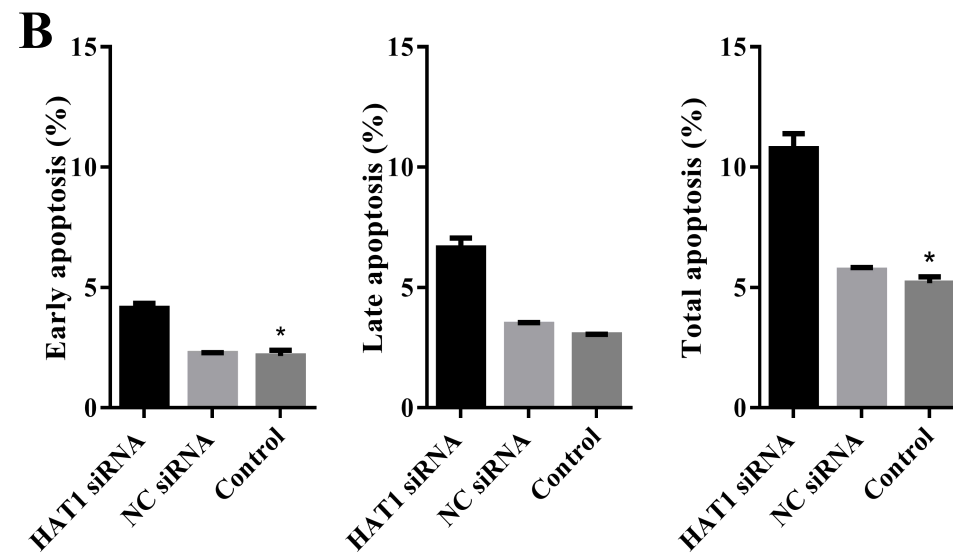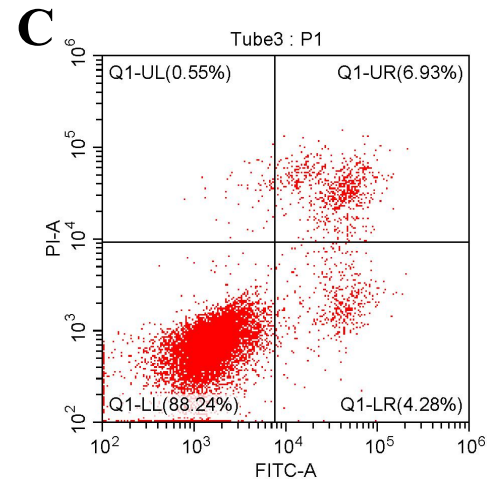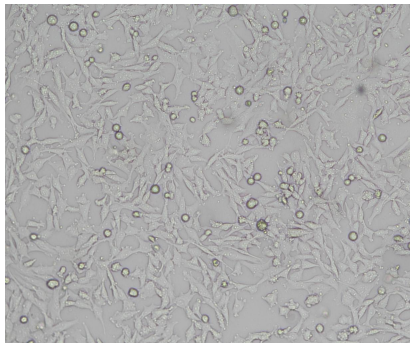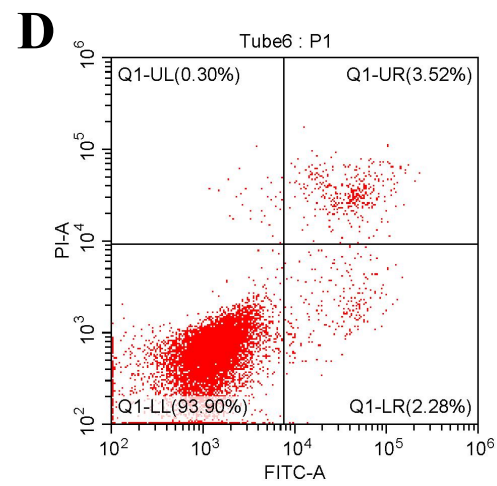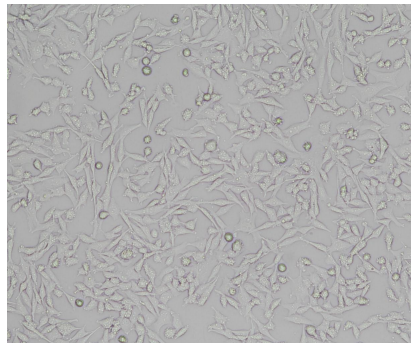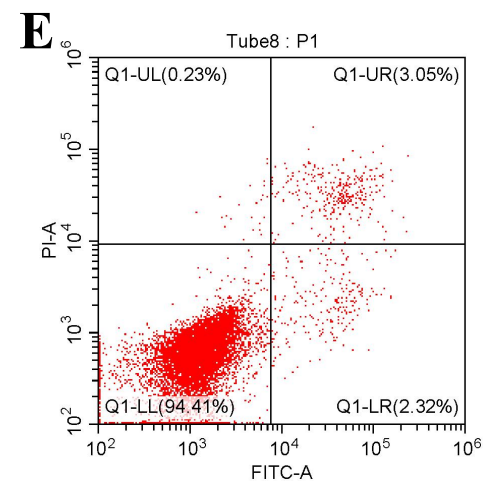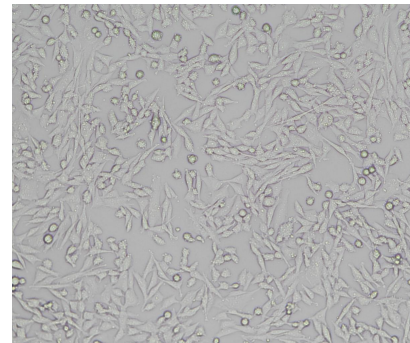

Supplement: S17 Fig — (A) Cell viability assessed by CCK-8 assay. DF-1 cells were transfected with HAT1 siRNA (50 nM), NC siRNA (50 nM), or mock control (transfection reagent alone). At 24, 48, and 72 h post-transfection, cell viability was measured and normalized to the control group. Data are presented as mean ± SEM from three independent experiments. (B) Quantitative analysis of apoptosis rates. Cells were harvested at 48 h post-transfection, stained with Annexin V/PI, and analyzed by flow cytometry. Percentages of early apoptotic (Annexin V ⁺ /PI⁻) and late apoptotic (Annexin V ⁺ /PI⁺) cells are shown. (C–E) Representative flow cytometry plots and corresponding phase-contrast micrographs of cells in the HAT1 siRNA group (C), NC siRNA group (D), and mock control group (E). (PDF) [file ppat.1014229.s021.pdf]
